# Inferring the genetic responses to acute drought stress across an ecological gradient

**DOI:** 10.1186/s12864-021-08178-w

**Published:** 2022-01-04

**Authors:** Jessica K. Devitt, Albert Chung, John J. Schenk

**Affiliations:** 1 Department of Biology, Georgia Southern University, Statesboro, GA 30460 USA; 2 Department of Ecology and Evolutionary Biology, University of California Los Angeles, Los Angeles, CA 90095-7246 USA; 3 Department of Environmental and Plant Biology, Ohio University, Athens, OH 457012979 USA

**Keywords:** Adaptive response, Differential gene expression, Drought, *Mentzelia*, Plant physiology, RNA-Seq, Transcriptome

## Abstract

**Background:**

How do xerophytic species thrive in environments that experience extreme annual drought? Although critical to the survival of many species, the genetic responses to drought stress in many non-model organisms has yet to be explored. We investigated this question in *Mentzelia* section *Bartonia* (Loasaceae), which occurs throughout western North America, including arid lands. To better understand the genetic responses to drought stress among species that occur in different habitats, the gene expression levels of three species from *Mentzelia* were compared across a precipitation gradient. Two de novo reference transcriptomes were generated and annotated. Leaf and root tissues were collected from control and drought shocked plants and compared to one another for differential expression. A target-gene approach was also implemented to better understand how drought-related genes from model and crop species function in non-model systems.

**Results:**

When comparing the drought-shock treatment plants to their respective control plants, we identified 165 differentially expressed clusters across all three species. Differentially expressed genes including those associated with water movement, photosynthesis, and delayed senescence. The transcriptome profiling approach was coupled with a target genes approach that measured expression of 90 genes associated with drought tolerance in model organisms. Comparing differentially expressed genes with a ≥ 2 log-fold value between species and tissue types showed significant differences in drought response. In pairwise comparisons, species that occurred in drier environments differentially expressed greater genes in leaves when drought shocked than those from wetter environments, but expression in the roots mostly produced opposite results.

**Conclusions:**

Arid-adapted species mount greater genetic responses compared to the mesophytic species, which has likely evolved in response to consistent annual drought exposure across generations. Drought responses also depended on organ type. Xerophytes, for example, mounted a larger response in leaves to downregulate photosynthesis and senescence, while mobilizing carbon and regulating water in the roots. The complexity of drought responses in *Mentzelia* suggest that whole organism responses need to be considered when studying drought and, in particular, the physiological mechanisms in which plants regulate water, carbon, cell death, metabolism, and secondary metabolites.

**Supplementary Information:**

The online version contains supplementary material available at 10.1186/s12864-021-08178-w.

## Background

Land plants have been adapting to drought stress since they first evolved onto land. Despite the potentially lethal consequences of drought stress, some plants only occur in arid regions that experience severe, annual drought. To cope with drought stress, plants have evolved morphological, metabolic, anatomical, and physiological adaptive responses [4, 17, 41]. Understanding the molecular processes that drive physiological responses in drought-stress adaptation is crucial as Earth’s climates become hotter and drier [4].

Although multiple studies have predicted ecosystem responses to instances of drought in model and agricultural species [4, 17, 28, 72, 73], non-model species have received much less attention even though they might respond differently when experiencing drought, which includes variation in the order of physiological responses, as well as the functional gene groups that differentially express in response to drought stress [28]. When drought-stress signals are received, signal transduction leads to the induction of both physiological and metabolic processes [38]. Consequently, molecular and physiological responses are usually linked, in which a change in gene expression causes a physiological change (e.g., stomatal closure). How species adapt to drought stress at the gene-expression level, however, is less well-characterized than the physiological processes they underlie, and could vary, or even converge, across plant groups.

The expression of genes associated with stress regulation plays an important role in drought response [41]. If plants regulate the impact of drought through molecular pathways, measuring the expression levels of genes that lead to physiological responses will illuminate how plants respond to stress in their natural environment. By discovering drought response genes, future studies will be able to distinguish how adaptive evolution in conjunction with physiological plasticity facilitates or constrains drought responses [14]. Although we have learned much from studies on how crop species and other model organisms respond to drought, less is known about how the remaining species of plants are able to cope with drought stress on a genetic level, especially those in xeric environments.

Plants have likely evolved numerous responses to mitigate drought stress, which includes genetic, physiological, and morphological adaptations. Environmental variation can play a further role, in which species that occur in xeric environments might respond differently to drought stress than those that occur in mesic environments. Although studies that have examined drought stress responses have shed much light on how a species responds to drought stress, experimental comparative studies of species across an environmental gradient have the potential to further tease apart how species adapt to drought stress. If we compare, for example, a group of closely related species that occur in xeric and mesic environments, hypotheses can be formulated and tested to determine how species have adapted genetic responses to drought stress.

We propose four hypotheses to explain the evolution of drought responses in a comparative framework. The first hypothesis (HA-1) proposes that xerophytes have a stronger genetic drought response than mesophytic species. If supported, we predict that xerophytes would have a significant difference in gene expression, whether up or down-regulated, whereas mesophytes would have no significant difference in gene expression. Hypothesis HA-1 suggests that the annual drought stress that xerophytes experience has selected for genetic responses and/or selection has not favored adaptive responses at the genetic level in mesophytes.

A second hypothesis (HA-2) posits that mesophytes have a stronger response when exposed to drought conditions than xerophytes. If supported, this would mean that mesophytes have a significant difference in gene expression, whether the difference is in genes that have been up or down-regulated, whereas xerophytes shows no significant difference in gene expression. Hypothesis HA-2 suggests that morphological adaptations (e.g., reduced leaf surface area to volume ratio) in xerophytes mitigate stress caused by drought.

A third hypothesis (HA-3) proposes that all species have similarly strong genetic responses when exposed to drought conditions regardless of their environment, suggesting that species that occur across an environmental gradient have inherited genetic responses to drought from an ancestor that itself adapted to drought conditions. If supported, hypothesis HA-3 might explain why multiple species of some clades have been successful in independently evolving into xeric environments.

Our final hypothesis is a null hypothesis (Ho) which proposes that all species fail to mount a genetic response to drought, exhibiting negligible differences in gene regulation. The null hypothesis, if supported, would suggest that plants do not respond to drought at the molecular level, rather, they have other means, such as morphological adaptations, or are susceptible to the consequences of drought stress.

Non-model plant systems might provide novel responses or mechanisms not identified in other model-plant systems, which could revolutionize the way crops are genetically modified for drought tolerance, in addition to adding to the knowledge of known genes and responses associated with drought. Because of sampling bias and potential undiscovered stress responses within plants, it is important to study species that are adapted across a wide range of habitats, including xeric ecosystems.

To test the above four hypotheses, we subjected populations from three species in the genus *Mentzelia* L. (Loasaceae) that occur across an environmental gradient to a drought shock experiment and compared them to non-drought-shocked individuals. The three species of *Mentzelia* occur naturally across a wide environmental gradient, from the southwestern North American deserts, to mesic habitats near the Continental Divide in the Rocky Mountains [60]. Some species of *Mentzelia* thrive in xeric habitats, but we know very little about how they mitigate drought stress other than having morphological adaptations associated with living in xeric environments that include leaves with reduced surface area and high trichome density [18]. To determine how *Mentzelia* responds to acute drought stress across an environmental gradient, we subjected three species to acute drought stress and measured and compared their gene expression levels.

## Results

### Quality assessment and annotation of assemblies

**Fig. 1:**
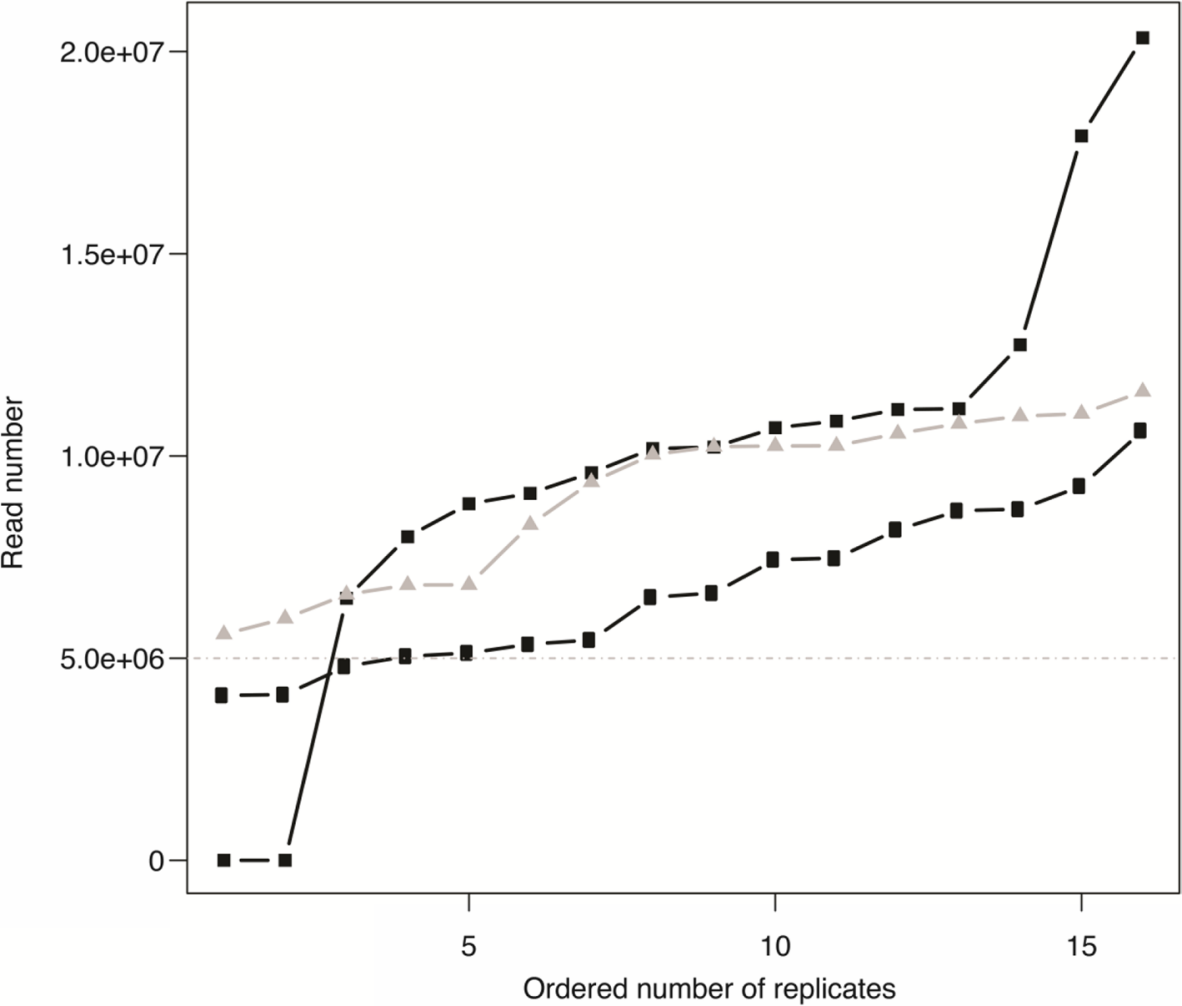
Number of reads per treatment per species for *Mentzelia reverchonii* (black squares), *M. filifolia* (black circles), and *M. speciosa* (gray triangles). X-axis is ordered from the smallest to the largest number or reads per treatment. Gray horizontal dashed line at 5 million reads represents an optimal minimum number of target reads needed to infer broad differential expression patterns

We sequenced approximately 35 Gb of pair-end read data from the cDNA libraries for eight replicates of two treatments of the xerophytic *M. filifolia*, the semi-arid *M. reverchonii*, and the mesic *M. speciosa*. We sequenced 4,079,266–20,339,791 reads per library, with an average read count of 9,004,449 per library (Fig. 1). Two replicates failed for *M. reverchonii*, one root and one leaf sample. All other sequences, except for three libraries from *M. filifolia* that produced 4,079,266, 4,099,930, and 4,800,251 reads, were above 5 million reads (Fig. 1). Average GC content for all samples of *Mentzelia filifolia* were 42.4% with 112,001 genes and 214,695 transcript assemblies. *Mentzelia speciosa* had an average GC content of 43.5%, with 143,880 genes and 259,156 transcript assemblies in total.

The quality and completeness of each reference was assessed using HISAT2 to map the original reads from each species back to the reference transcriptomes. The average alignment rate for *M. filifolia* was 84.7% (Table 1), and the average alignment rate for *M. speciosa* was 85.4% (Table 2). The Trinotate annotation report for *M. filifolia* showed 92,821 known sequence hits using Swiss-Prot annotation. Approximately 52,843 known GO terms were annotated along with 47,088 known pathways using KEGG pathway enrichment analysis, and 44,275 transcript annotations using EggNOG (Table 3). The Trinotate annotation report for *M. speciosa* showed 105,007 known sequence hits using Swiss-Prot annotation along with 60,454 GO, 53,234 KEGG, and 43,701 EggNOG annotations. BUSCO analyses were performed to determine the completeness of each de novo assembled transcriptome using evolutionarily known gene content from universal single copy orthologs, therefore providing a measure of whether our sequencing coverage adequately sequenced transcriptomes. The transcriptomes were sufficiently sequenced, as 94.1% of the surveyed genes included in the assembly of *M. filifolia* were found to be complete single or duplicated genes, while 94.7% of genes surveyed from the *M. speciosa* assembly were found to be complete single or duplicated genes (Table 4).

**Table 1:** Number of sequencing reads and alignment rate percentages calculated using HISAT2 for samples of *Mentzelia filifolia*. Root and leaf tissues are represented using “R” and “L” in the sample names

Sample	Number of reads	Alignment rate (%)
2502_STEM	6,375,918	88.78
2502_FRUIT	6,029,059	77.9
2502_FLOWER	5,857,379	92.99
2501_R2	10,627,333	89.7
2501_L1	8,175,813	81.7
2500_R3	4,800,251	89.63
2500_L2	9,254,149	83.2
2499_R2	8,682,337	84.56
2499_L2	8,647,148	86.58
2498_R2	6,510,285	82.37
2498_L2	6,612,024	81.01
2496_R1	5,348,465	84.82
2496_L2	5,128,545	87.58
2495_R1	5,048,690	87.96
2495_L2	7,435,690	85.16
2494_R1	4,099,930	80.07
2494_L1	4,079,266	77.97
2493_R1	7,471,170	80.58
2493_L1	5,450,266	86.02
Average:	6,612,300.94	84.66

**Table 2:** Number of sequencing reads and alignment rate percentages calculated using HISAT2 for samples of *Mentzelia speciosa.* Root and leaf tissues are represented using “R” and “L” in the sample names

Sample	Number of reads	Alignment rate (%)
001_R3	8,295,850	86.38
001_L1	10,983,630	88.64
002_R1	5,982,619	88.39
002_R3	10,553,058	87.53
003_L1	9,353,240	88.15
003_R3	10,251,241	80.62
004_L1	6,813,629	85.69
004_R3	6,811,305	88.27
006_L1	5,591,754	73.00
006_R3	6,575,857	87.92
007_L1	10,246,401	86.77
007_R3	10,797,019	89.76
008_L1	11,591,576	85.29
008_R3	11,046,039	92.32
009_L1	10,033,878	89.23
009_R2	10,228,942	87.18
011_FLOWER	5,938,309	87.66
012_FRUIT	270	65.74
013_STEM	7,647,140	83.46
Average	8,354,829.31	85.36

**Table 3:** Annotation summary for the de novo assembly of each *Mentzelia filifolia* and *M. speciosa*

Annotation category	*M. filifolia*	*M. speciosa*
Annotated genes	116,537	122,940
Transcripts with Swiss-Prot annotation	92,821	105,007
Transcripts with KEGG annotation	47,088	53,234
Transcripts with GO annotation	52,843	60,454
Transcripts with EggNOG annotation	44,275	43,701

**Table 4:** BUSCO results for the de novo assembled transcriptomes showing quantitative measures for each transcriptome assembly of *Mentzelia filifolia* and *M. speciosa* based on evolutionarily informed expectations of gene content

BUSCO category	*M. filifolia*	*M. speciosa*
Complete genes	94.10%	94.7%
Complete single-copy genes	34.70%	26.7%
Complete duplicated genes	59.40%	68.0%
Fragmented genes	4.60%	4.60%
Missing	1.30%	0.07%

### Differential expression in roots and leaves

**Fig. 2:**
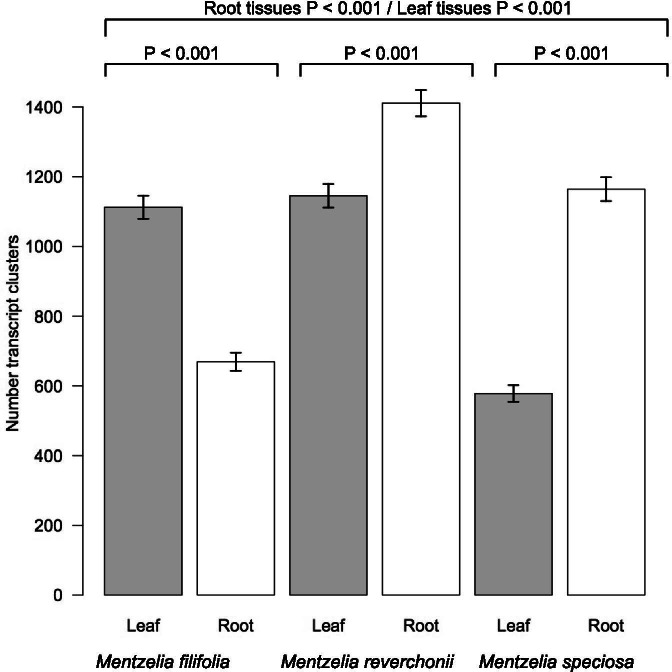
Number of differentially expressed transcript clusters with a logFC ≥2 from each species and tissue type resulting from the differential expression analysis in edgeR. Significant relationships between species and tissue types are designated by bars showing differences between tissues within a single species as well as differences between tissue types across species

Among the 24 leaf and 24 root tissues sampled from three species, corset produced 64,858 leaf and 70,468 root transcript-clusters for *M. filifolia*, 82,205 leaf and 59,173 root transcript-clusters for *M. reverchonii*, and 54,292 leaf and 93,140 root transcript-clusters for *M. speciosa.* When considering differentially expressed (DE) genes with a log fold-change (logFC) ≥ 2, the results identified 6079 DE genes. *Mentzelia filifolia* leaves had 1112 DE genes, while roots had 669 DE genes. *Mentzelia reverchonii* had 1145 DE genes in leaves and 1411 in roots. *Mentzelia speciosa* resulted in 578 DE genes in leaves and 1164 in roots (Fig. 2).

**Fig. 3:**
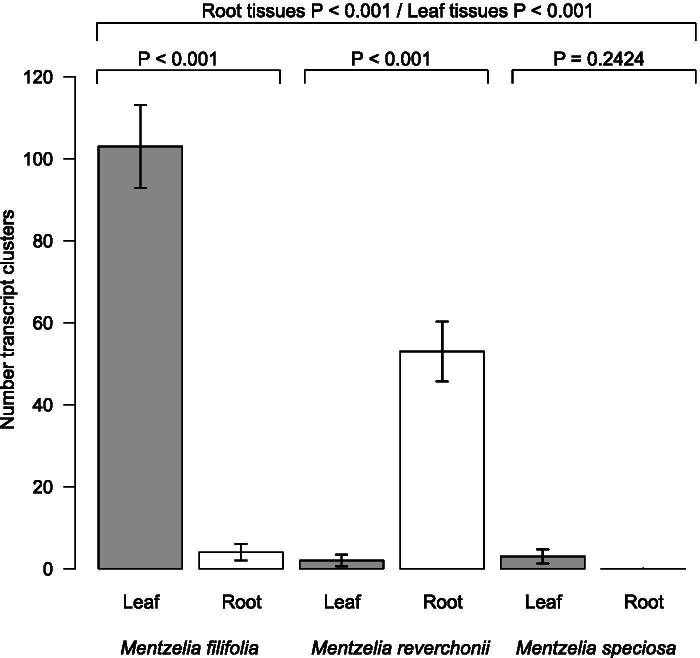
Number of significant (false discovery rate ≤ 0.05) differentially expressed genes from each species and tissue type resulting from the differential expression analysis using edgeR

The DE analyses with a false discovery rate *P*-value adjustment resulted in 165 DE cluster-transcripts across all species and tissue types, and among them, 140 were identified to homologs (Fig. 3). The 25 remaining DE cluster transcripts were not assigned to homologous sequences and, therefore, not analyzed further. *Mentzelia filifolia* differentially expressed 103 transcripts in leaf and four in root tissues. Nine transcript-clusters were significantly up-regulated in *M. filifolia* leaves and 94 were significantly down-regulated (Table 5). The most down- regulated genes were Adenylate cyclase proteins, which were reduced by a logFC of − 8.4. Up-regulated transcript-clusters were categorized as endonuclease activity, wound response, membrane components, and nitrate reductase (NADH) activity. A differential expression analysis of roots from *M. filifolia* produced four down-regulated transcript-clusters (Table 6).

**Table 5:** Differentially expressed genes in leaf tissue of *Mentzelia filifolia* from the drought-shocked treatment compared to the control. Measures of log-fold change (logFC) and false discovery rate (FDR) were used to determine significance and direction of regulation. LogFC values are in comparison to the control levels of expression from the same tissue/species

Transcript cluster ID	Annotation	logFC	FDR
Cluster-58816.0	PREDICTED: uncharacterized protein LOC104428360	-8.37	0.0028
Cluster-48670.0	Protein TAR1	-7.81	0.0013
Cluster-58105.0	Hypothetical protein CDL37_04775, partial	-7.72	0.0011
Cluster-39297.0	Hypothetical protein CDL37_04775, partial	-7.60	0.0011
Cluster-50655.1	Hypothetical protein T12_6945, partial	-7.59	0.0011
Cluster-41545.0	PREDICTED: uncharacterized protein LOC109358019	-7.52	0.0011
Cluster-60839.0	Protein TAR1	-7.49	0.0011
Cluster-34501.0	Hypothetical protein BC332_34878	-7.49	0.0011
Cluster-62161.0	Predicted protein	-7.44	0.0015
Cluster-34654.0	Protein TAR1	-7.40	0.0011
Cluster-7656.2	Transmembrane protein, putative	-7.38	0.0011
Cluster-41578.0	Hypothetical protein BC332_34878	-7.36	0.0011
Cluster-41545.1	PREDICTED: uncharacterized protein LOC106795708	-7.35	0.0011
Cluster-50091.0	Protein TAR1	-7.30	0.0011
Cluster-49212.0	ATP synthase subunit beta	-7.29	0.0011
Cluster-31834.0	Protein TAR1	-7.28	0.0024
Cluster-49252.0	Hypothetical protein GLYMA_13G0132002, partial	-7.23	0.0054
Cluster-34739.0	Predicted protein	-7.22	0.0013
Cluster-36863.0	Regulator of rDNA transcription protein 15	-7.20	0.0011
Cluster-47860.0	Protein TAR1	-7.19	0.0011
Cluster-40814.0	Protein TAR1	-7.19	0.0018
Cluster-52445.0	Regulator of rDNA transcription protein 15	-7.19	0.0011
Cluster-35862.0	Uncharacterized protein LOC110824766, partial	-7.18	0.0018
Cluster-51124.0	Alpha-L1 nicotinic acetyl choline receptor	-7.14	0.0011
Cluster-59385.0	Hypothetical protein CQW23_33511	-7.14	0.0011
Cluster-37035.0	Cytochrome P450 monooxygenase	-7.12	0.0015
Cluster-57663.0	Predicted protein	-7.11	0.0011
Cluster-51272.0	Regulator of rDNA transcription protein 15	-7.10	0.0011
Cluster-60616.1	Hypothetical protein CQW23_33755	-7.08	0.0018
Cluster-59060.0	Predicted protein	-7.02	0.0011
Cluster-52045.0	Cytochrome P450 like_TBP	-7.01	0.0011
Cluster-23459.2	Regulator of rDNA transcription protein 15	-7.00	0.0011
Cluster-42269.0	Protein TAR1	-6.97	0.0011
Cluster-62261.0	Predicted protein	-6.93	0.0011
Cluster-36057.0	CASP-like protein 4A3	-6.91	0.0011
Cluster-38658.0	Protein TAR1	-6.87	0.0011
Cluster-59522.0	Transmembrane protein, putative	-6.84	0.0011
Cluster-63249.0	Aquaporin TIP1-1	-6.52	0.0011
Cluster-55831.0	Protein TAR1-like	-6.52	0.0016
Cluster-41007.0	Regulator of rDNA transcription protein 15	-6.47	0.0021
Cluster-51769.0	Regulator of rDNA transcription protein 15	-6.37	0.0016
Cluster-56791.0	Uncharacterized protein LOC109940280	-6.37	0.0032
Cluster-50338.0	Hypothetical protein BUMB_02141	-6.31	0.0023
Cluster-31006.0	Regulator of rDNA transcription protein 15	-6.24	0.0012
Cluster-59353.0	Regulator of rDNA transcription protein 15	-6.06	0.0018
Cluster-50764.0	Hypothetical protein T459_27227	-6.04	0.0051
Cluster-51946.0	Regulator of rDNA transcription protein 15	-6.04	0.0034
Cluster-60097.0	Regulator of rDNA transcription protein 15	-5.89	0.0029
Cluster-42721.0	Regulator of rDNA transcription protein 15	-5.87	0.0028
Cluster-56732.0	No homology	-5.72	0.0067
Cluster-35355.0	Cytochrome P450-like TBP protein	-5.59	0.0137
Cluster-46825.0	Regulator of rDNA transcription protein 15	-5.57	0.0032
Cluster-53270.0	Hypothetical protein CQW23_33511	-5.40	0.0052
Cluster-44046.0	Probable inactive patatin-like protein 9	-5.06	0.0054
Cluster-32710.0	PREDICTED: uncharacterized protein LOC104811909	-5.04	0.0149
Cluster-34807.0	Zinc finger protein 1	-4.99	0.0189
Cluster-59670.0	Senescence-associated protein, putative	-4.97	0.0193
Cluster-41530.0	Hypothetical protein SERLA73DRAFT_67532, partial	-4.93	0.0011
Cluster-51948.0	Hypothetical protein GLYMA_13G013900	-4.91	0.0016
Cluster-61489.1	Predicted protein	-4.80	0.0016
Cluster-53940.0	Wound-responsive family protein	-4.79	0.0331
Cluster-57103.0	Delta(24)-sterol reductase	-4.73	0.0185
Cluster-31264.0	Hypothetical protein OXYTRI_14248 (macronuclear)	-4.70	0.0016
Cluster-61489.2	Hypothetical protein GLYMA_13G015500, partial	-4.65	0.0018
Cluster-60530.0	Probable inactive receptor kinase At5g67200	-4.55	0.0365
Cluster-38796.0	Uncharacterized protein LOC110277292	-4.54	0.0025
Cluster-62327.0	Uncharacterised protein	-4.47	0.0388
Cluster-61719.0	Organ-specific protein S2-like	-4.43	0.0445
Cluster-53551.0	Tar1p like protein	-4.38	0.0022
Cluster-55762.0	Tar1p, partial	-4.35	0.0140
Cluster-42910.0	PREDICTED: uncharacterized protein LOC105736981	-4.33	0.0031
Cluster-62007.0	Hypothetical protein GLYMA_U007300	-4.27	0.0113
Cluster-35209.0	No homology	-4.23	0.0194
Cluster-42867.0	Acidic endochitinase-like	-4.22	0.0462
Cluster-50876.0	Indole-3-acetic acid-induced protein ARG2-like	-4.16	0.0392
Cluster-48507.0	Regulator of rDNA transcription protein 15	-4.15	0.0392
Cluster-57366.0	No homology	-4.10	0.0053
Cluster-52390.3	Metallocarboxypeptidase inhibitor	-4.07	0.0123
Cluster-63044.0	Hypothetical protein GOBAR_DD19277	-4.05	0.0251
Cluster-54706.0	S-adenosylmethionine decarboxylase proenzyme-like	-3.99	0.0270
Cluster-36407.0	Hypothetical protein GLYMA_U007300	-3.96	0.0093
Cluster-59427.0	Hypothetical protein SERLA73DRAFT_67532, partial	-3.91	0.0073
Cluster-41436.2	Chromosome 3B, genomic scaffold	-3.89	0.0117
Cluster-61466.0	rRNA intron-encoded homing endonuclease, putative	-3.86	0.0251
Cluster-61875.0	rRNA intron-encoded homing endonuclease	-3.86	0.0194
Cluster-51170.0	UNKNOWN	-3.59	0.0295
Cluster-34949.0	Indole-3-acetate O-methyltransferase 1	-3.56	0.0270
Cluster-59063.0	PREDICTED: uncharacterized protein LOC109176672	-3.54	0.0178
Cluster-42283.0	Senescence-associated protein	-3.48	0.0416
Cluster-34872.0	Cold-regulated protein	-3.37	0.0270
Cluster-43131.0	Tonoplast dicarboxylate transporter	-3.27	0.0445
Cluster-34593.0	No homology	-3.21	0.0331
Cluster-54555.0	No homology	-2.94	0.0404
Cluster-47071.0	Hypothetical protein ABT39_MTgene6262	2.98	0.0451
Cluster-61726.0	No homology	3.25	0.0138
Cluster-40722.0	No homology	3.78	0.0335
Cluster-62217.0	Protein RADIALIS-like 5	3.98	0.0375
Cluster-52889.0	Nitrate reductase 2	4.13	0.0331
Cluster-33868.0	Crocetin glucosyltransferase, chloroplastic-like	4.32	0.0171
Cluster-63829.0	No homology	6.40	0.0024
Cluster-48746.0	No homology	7.00	0.0025
Cluster-33695.0	Probable xyloglucan endotransglucosylase/Hydrolase protein 23	-4.54	0.0080
Cluster-58851.0	Pentatricopeptide repeat-containing protein At1g07590, Mitochondrial	3.64	0.0134

**Table 6:** Differentially expressed genes in root tissue of *Mentzelia. filifolia* from the drought-shocked treatment compared to the control. Measures of the log-fold change (logFC) and false discovery rate (FDR) were used to determine significance and direction of regulation

Transcript cluster ID	Annotation	logFC	FDR
Cluster-33699.0	Transcript antisense to ribosomal RNA protein	9.99	0.0153
Cluster-65674.0	CYP76A26-like protein	−4.92	0.0153
Cluster-60975.0	No homology	8.08	0.0153
Cluster-49634.0	No homology	7.23	0.0164

*Mentzelia reverchonii* differentially expressed two transcripts in leaf and 53 in roots. The DE analysis of leaves resulted in two over-expressed transcript-clusters, each categorized as being involved in the oxidation reduction process (Table 7). There were 28 down-regulated and 25 up-regulated transcript-clusters in the roots. The most highly up-regulated cluster was expressed with 7.0 logFC and was categorized as participating in ubiquitin-protein transferase activity and protein ubiquitination (Table 8).

**Table 7:** Differentially expressed genes in leaf tissue of *Mentzelia reverchonii* from the drought-shocked treatment compared to the control. Measures of the log-fold change (logFC) and false discovery rate (FDR) were used to determine significance and direction of regulation

Transcript cluster ID	Annotation	logFC	FDR
Cluster-49973.1	Ribulose bisphosphate carboxylase small chain	8.98	0.0154
Cluster-24541.0 l	Ong-chain-alcohol oxidase FAO2-like	4.25	0.0167

**Table 8:** Differentially expressed genes in root tissue from *Mentzelia reverchonii* from the drought-shocked treatment compared to the control. Measures of the log-fold change (logFC) and false discovery rate (FDR) were used to determine significance and direction of regulation

Transcript cluster ID	Annotation	logFC	FDR
Cluster-38908.0	Remorin-like isoform X2	6.30	0.0152
Cluster-49883.0	NAC domain-containing protein 72	4.75	0.0152
Cluster-55672.0	GATA transcription factor 8-like	4.33	0.0152
Cluster-18781.0	21 kDa protein-like	6.65	0.0223
Cluster-36496.0	Non-specific lipid transfer protein GPI-anchored 2-like	4.45	0.0224
Cluster-35195.0	PREDICTED: uncharacterized protein LOC108988160	−4.56	0.0224
Cluster-51946.0	No homology	−4.31	0.0224
Cluster-25227.0	U-box domain-containing protein 19	7.00	0.0224
Cluster-27607.0	Squamosa promoter-binding protein 1-like	4.65	0.0224
Cluster-35437.0	CHK1 checkpoint-like protein	−10.93	0.0224
Cluster-56684.0	No homology	−4.69	0.0224
Cluster-15975.0	PREDICTED: uncharacterized protein LOC100854478	3.61	0.0224
Cluster-20450.0	REF/SRPP-like protein At3g05500	5.96	0.0224
Cluster-41375.0	Cellulose synthase A catalytic subunit 2	5.04	0.0224
Cluster-16220.0	No homology	5.34	0.0224
Cluster-47851.0	No homology	3.78	0.0289
Cluster-17790.0	Acyl-lipid (9–3)-desaturase-like	3.77	0.0289
Cluster-51094.0	Unknown	−5.75	0.0289
Cluster-57461.0	Ubiquitin-like protein	3.35	0.0320
Cluster-25176.0	Pollen-specific protein SF21-like	3.72	0.0320
Cluster-38124.0	L-ascorbate oxidase homolog	5.81	0.0320
Cluster-34320.0	No homology	−4.14	0.0331
Cluster-49004.0	No homology	4.83	0.0347
Cluster-30840.0	Two-pore potassium channel 1-like	4.93	0.0347
Cluster-20850.0	Sodium/calcium exchanger NCL-like	5.30	0.0347
Cluster-37384.0	Sodium/calcium exchanger NCL-like	3.91	0.0357
Cluster-48594.0	Atp synthase subunit beta	−7.03	0.0362
Cluster-11814.0	No homology	−3.32	0.0362
Cluster-39132.0	Inositol-tetrakisphosphate 1-kinase 1-like	4.30	0.0381
Cluster-38020.0	No homology	−3.35	0.0440
Cluster-47551.0	10 kDa putative secreted protein	−6.65	0.0440
Cluster-55259.0	No homology	−3.92	0.0440
Cluster-33758.0	Hypothetical protein X975_24482, partial	−7.16	0.0453
Cluster-25182.0	Glucose repressible protein Grg1	−5.13	0.0453
Cluster-23747.0	Predicted protein	−7.41	0.0453
Cluster-23745.0	Probable WRKY transcription factor 75	−3.18	0.0453
Cluster-24748.0	Protein SRC2-like	3.81	0.0453
Cluster-51512.0	Predicted protein	−6.41	0.0453
Cluster-5078.0	Late embryogenesis abundant protein	4.99	0.0453
Cluster-53464.0	Putative oRF58e	−9.37	0.0453
Cluster-41067.0	UNKNOWN	−7.99	0.0453
Cluster-7120.0	Hypothetical protein V565_194550, partial	−10.29	0.0467
Cluster-17962.0	No homology	−8.68	0.0467
Cluster-27793.0	Homeobox protein SBH1	−5.01	0.0467
Cluster-23243.0	17.3 kDa class I heat shock protein-like	5.14	0.0475
Cluster-22949.0	PG1 protein, homology to *Homo sapiens*	−8.80	0.0477
Cluster-56300.0	No homology	−5.63	0.0488
Cluster-24206.0	Extradiol ring-cleavage dioxygenase-like	−2.75	0.0494
Cluster-33282.0	No homology	−3.41	0.0496
Cluster-36817.1	No homology	−3.60	0.0496
Cluster-16449.0	Uncharacterised protein	−8.93	0.0496
Cluster-42928.0	Uncharacterized aarF domain-containing protein kinase At1g79600, chloroplastic	−3.68	0.0224
Cluster-36934.0	Sec-independent protein translocase protein TATC, Chloroplastic	6.05	0.0374

*Mentzelia speciosa* differentially expressed three transcripts in leaf and none in root tissue. Two clusters were up-regulated and a single transcript-cluster was down-regulated (Table 9).

**Table 9:** Differentially expressed genes in leaf tissue from *Mentzelia speciosa* from the drought-shocked treatment compared to the control. Measures of the log-fold change (logFC) and false discovery rate (FDR) were used to determine significance and direction of regulation

Transcript cluster ID	Annotation	logFC	FDR
Cluster-38,359.0	Polyubiquitin-like	7.25	0.0141
Cluster-44,349.15	Glucose repressible protein Grg1	8.49	0.0396
Cluster-27,554.0	Uncharacterized protein	−4.60	0.0396

**Fig. 4:**
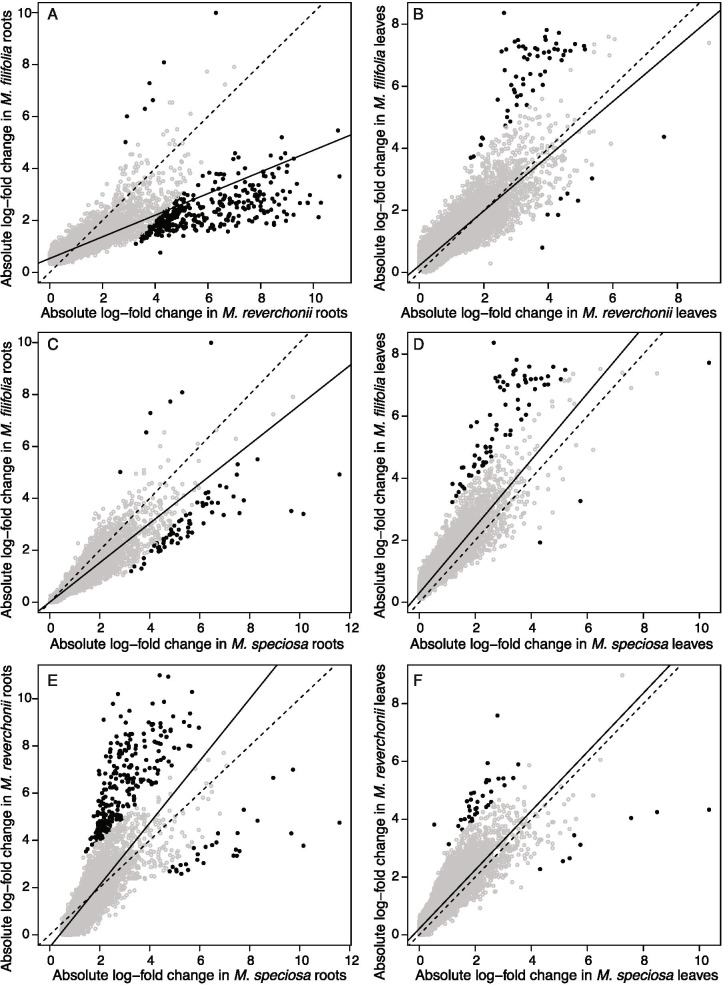
Pairwise comparisons of differentially expressed genes in the drought-shock treatment among the tissue types of the focal species. Each point represents the absolute expression level of a gene/cluster for both species being compared. The dashed black line is the expected line (slope = 1) if the two species expressed all loci at identical levels and informs whether one species/tissue is mounting a greater response than the other. The solid black line is the best fit line for the data given a linear model and tells us where the majority of the points lay. All solid black slopes were significantly different than 1 at alpha = 0.05 (*P* < 0.001). The grey open circles indicate a log-fold change less than 2, and the solid black circles indicate a log-fold change of 2 or greater. In all comparisons, the species that occurs in the more arid habitat is placed on the y-axis

The xerophytic *M. filifolia* had a stronger response than mesophytic *M. speciosa* and the intermediate semi-arid *M. reverchonii* when comparing gene response in leaves overall (Fig. 4). When comparing roots, however, *M. speciosa* and *M. reverchonii* had a greater response to drought compared to *M. filifolia* (Fig. 4A, C). *Mentzelia reverchonii* produced a greater response in both leaf and root tissues when compared to *M. speciosa* (Fig. 4E, F), while *M. filifolia* produced a greater response in leaves only compared to *M. speciosa*. Overall, *M. speciosa* did not mount as large of a response to drought shock compared to the xeric *M. filifolia* and semi-arid *M. reverchonii*.

### Target gene expression analysis

**Fig. 5:**
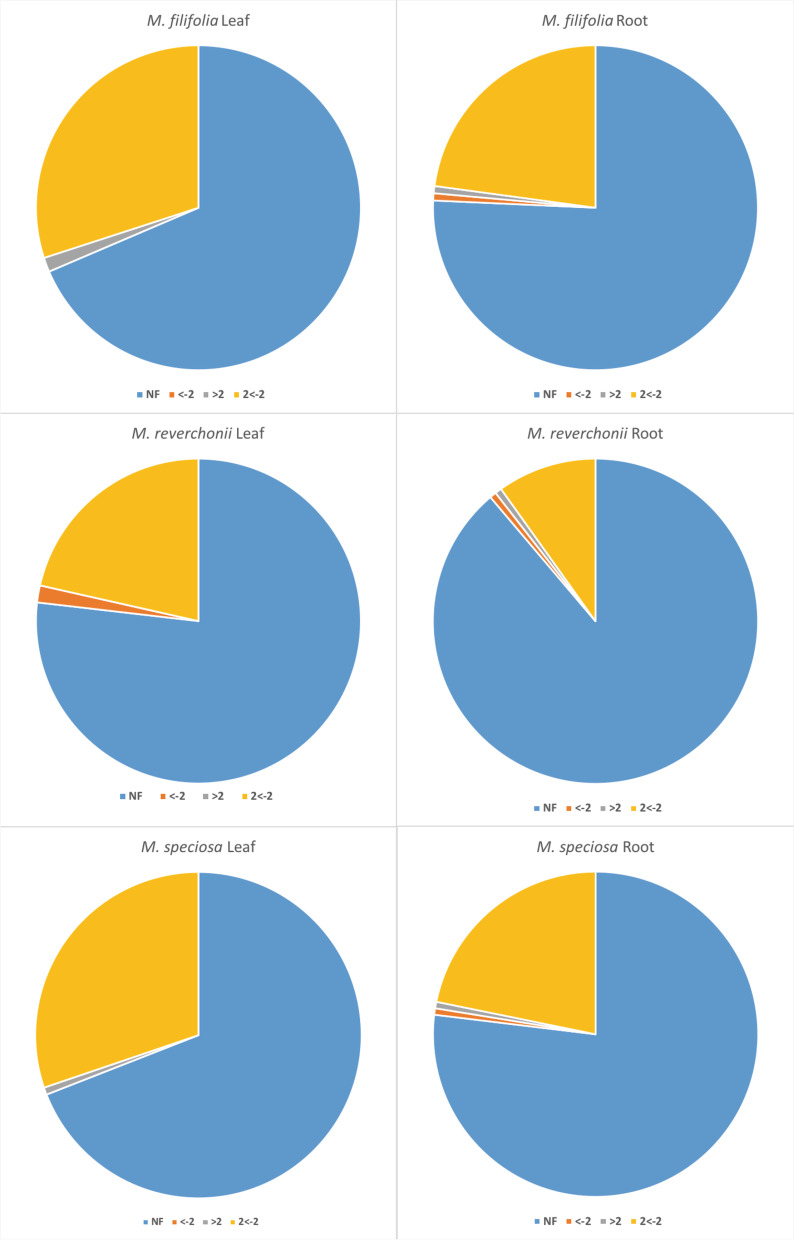
Proportion of target genes categorized into levels of expression by log-fold change. Each species and tissue type is represented in a single pie chart showing target genes with biologically meaningful log-fold changes. Genes that are expressed at a level less than − 2 logFC are displayed in red, greater than 2 logFC in gray, between − 2 and 2 logFC in yellow, with all target genes that were not expressed in blue

The target gene analysis included 90 target genes (Tables 10, 11). Because multiple transcript-clusters were identified as functions from the same process through GO annotating, multiple transcript-clusters associated with a single target gene. In total, 140 clusters matched to the targeted genes in *M. filifolia* leaves and 44 out of 140 clusters were included in the edgeR exact test analysis and were not filtered out before the analysis. Using a target gene approach, we compared co-expression of genes across species. From the 12 target genes that resulted in a logFC ≥2, we compared how they are expressed similarly between the three species and tissue types. Results are summarized in Fig. 5, Tables 2, 4, 5, 6, 7, 8, 9, 10 and 11.

**Table 10:** Set of target genes selected from previous studies that were determined to respond to drought

Entry	Entry name	Protein name
Q6ZKC0	14333_ORYSJ	14–3-3-like protein GF14-C
Q9SGW3	PSD8A_ARATH	26S proteasome non-ATPase regulatory subunit
Q9SIB2	KCS12_ARATH	3-ketoacyl-CoA synthase 12
Q9LRR7	NCED3_ARATH	9-cis-epoxycarotenoid dioxygenase NCED3
Q9FH76	ABAH3_ARATH	Abscisic acid 8′-hydroxylase 3
O80920	PYL4_ARATH	Abscisic acid receptor PYL4
Q9FLB1	PYL5_ARATH	Abscisic acid receptor PYL5
Q9FGM1	PYL8_ARATH	Abscisic acid receptor PYL8
Q9M7Q4	AI5L5_ARATH	ABSCISIC ACID-INSENSITIVE 5-like protein 5
Q9FXT4	AGAL_ORYSJ	Alpha-galactosidase
Q39958	Q39958_HELAN	Aquaporin
P43286	PIP21_ARATH	Aquaporin PIP2–1
Q9SI64	SPE1_ARATH	Arginine decarboxylase 1
Q9FT74	RQL1_ARATH	ATP-dependent DNA helicase Q-like 1
Q0WVW7	RQL5_ARATH	ATP-dependent DNA helicase Q-like 5
Q10D00	SUV3M_ORYSJ	ATP-dependent RNA helicase SUV3
P93022	ARFG_ARATH	Auxin response factor 7
Q9SRU2	BIG_ARATH	Auxin transport protein BIG
Q96247	AUX1_ARATH	Auxin transporter protein 1
Q6IVL3	Q6IVL3_GOSHI	C-repeat binding factor 15
Q9M101	CDPKN_ARATH	Calcium-dependent protein kinase 23
Q9S7J7	CB22_ARATH	Chlorophyll a-b binding protein 2.2
Q9S7M0	CB3_ARATH	Chlorophyll a-b binding protein 3, chloroplastic
P27521	CA4_ARATH	Chlorophyll a-b binding protein 4
O82132	DRE2A_ARATH	Dehydration-responsive element-binding protein 2A
Q9M0L0	DRE1A_ARATH	Dehydration-responsive element-binding protein 1A
P93835	DRE1B_ARATH	Dehydration-responsive element-binding protein 1B
Q9SYS6	DRE1C_ARATH	Dehydration-responsive element-binding protein 1C
O82132	DRE2A_ARATH	Dehydration-responsive element-binding protein 2A
P31168	COR47_ARATH	Dehydrin COR47
Q94AK4	RZF1_ARATH	E3 ubiquitin-protein ligase RZF1
Q9M2S6	SDIR1_ARATH	E3 ubiquitin-protein ligase SDIR1
A0A1S2Z179	A0A1S2Z179_CICAR	E3 ubiquitin-protein ligase SDIR1-like
Q84JL3	SINA3_ARATH	E3 ubiquitin-protein ligase SINAT3
Q9FNA4	ELP1_ARATH	Elongator complex protein 1
A0MES8	ABI4_ARATH	Ethylene-responsive transcription factor ABI4
Q9XI33	WIN1_ARATH	Ethylene-responsive transcription factor WIN1
P22197	ALFC7_ARATH	Fructose-bisphosphate aldolase 7
O80518	GOLS3_ARATH	Galactinol synthase 3
P42761	GSTFA_ARATH	Glutathione S-transferase F10
Q9ZRW8	GSTUJ_ARATH	Glutathione S-transferase U19
Q9C9W5	HPR1_ARATH	Glycerate dehydrogenase HPR, peroxisomal (GDH)
Q9M8L4	GLPK_ARATH	Glycerol kinase (Glycerokinase)
Q9LSV0	GLYR1_ARATH	Glyoxylate/succinic semialdehyde reductase 1
Q9LD83	SLAC1_ARATH	Guard cell S-type anion channel SLAC1
Q9SXL4	AHK1_ARATH	Histidine kinase 1
Q9C5U1	AHK3_ARATH	Histidine kinase 3
Q9C5U0	AHK4_ARATH	Histidine kinase 4
Q8L9T7	AHP5_ARATH	Histidine-containing phosphotransfer protein 5
Q8VZ59	YUC6_ARATH	Indole-3-pyruvate monooxygenase YUCCA6
B6UH99	B6UH99_MAIZE	Late embryogeneis abundant protein Lea14-A
Q9M0X3	Q9M0X3_ARATH	Late embryogenesis abundant
F4JQF1	F4JQF1_ARATH	Late embryogenesis abundant
Q9FG31	LEA46_ARATH	Late embryogenesis abundant protein 46
Q39084	LEA41_ARATH	Late embryogenis abundant protein
Q9XIA9	LACS2_ARATH	Long chain acyl-CoA synthetase 2
Q9SMX3	VDAC3_ARATH	Mitochondrial outer membrane protein porin 3
O81845	PUMP1_ARATH	Mitochondrial uncoupling protein 1 (AtPUMP1)
Q94A06	M2K1_ARATH	Mitogen-activated protein kinase kinase 1
O81472	MP3K2_ARATH	Mitogen-activated protein kinase kinase kinase 9
Q9SQY0	NAC52_ARATH	NAC domain containing protein 52
Q9XIN7	NAC40_ARATH	NAC domain-containing protein 40
Q7F2L3	NAC48_ORYSJ	NAC domain-containing protein 48
Q949N0	NAC53_ARATH	NAC domain-containing protein 53
Q9SCK6	NAC62_ARATH	NAC domain-containing protein 62
Q9LD44	NAC56_ARATH	NAC transcription factor 56
Q0PGJ6	AKRC9_ARATH	NADPH-dependent aldo-keto reductase
Q9SRQ7	NPC4_ARATH	Non-specific phospholipase C4
Q5U9M2	Q5U9M2_ORYSJ	Ornithine decarboxylase
Q0J265	Q0J265_ORYSJ	Os09g0375300 protein
P24101	PER33_ARATH	Peroxidase 33
Q9SMU8	PER34_ARATH	Peroxidase 34
Q8H112	PGL1A_ARATH	PGR5-like protein 1A
Q9FND9	RFS5_ARATH	Probable galactinol--sucrose galactosyltransferase 5
Q9SJN0	ABI5_ARATH	Protein ABSCISIC ACID-INSENSITIVE 5
Q9SFB0	DTX43_ARATH	Protein DETOXIFICATION 43
Q94BS2	MET1_ARATH	Protein MET1
Q7XJ04	Q7XJ04_ORYSJ	Putative ornithine decarboxylase
Q9LTX3	PPOX1_ARATH	Pyridoxine/pyridoxamine 5′-phosphate oxidase 1
P22200	KPYC_SOLTU	Pyruvate kinase, cytosolic isozyme
O48791	SCAB1_ARATH	Stomatal closure-related actin-binding protein 1
O82663	SDHA1_ARATH	Succinate dehydrogenase flavoprotein subunit 1
Q39232	SUC1_ARATH	Sucrose transport protein SUC1
Q9LNV3	STP2_ARATH	Sugar transport protein 2
Q24JK1	MYB96_ARATH	Transcription factor MYB96
Q9SNC6	PUB13_ARATH	U-box domain-containing protein 13
Q8RWG1	AB1K1_ARATH	Protein activity of BC1 complex kinase 1
Q39096	ERD15_ARATH	Protein early responsive to dehydration 15
Q9FGI6	NDUS1_ARATH	NADH dehydrogenase [ubiquinone] iron-sulfur protein 1
Q7XYY2	MED25_ARATH	Mediator of RNA polymerase II transcription subunit 25

**Table 11:** Differentially expressed target genes with a log-fold change (logFC) ≥ 2 found in each species of *Mentzelia* and tissue type

Species/tissue	Target gene	LogFC	*P*-value	Description
*M. filifolia* leaf	COR47_ARATH	2.105	0.008	Dehydrin COR47
	ERD15_ARATH	2.031	0.031	Protein early responsive to dehydration 15
*M. filifolia* root	NAC56_ARATH	2.896	0.033	NAC transcription factor 56
	PGL1A_ARATH	−3.470	0.024	PGR5-like protein 1A
*M. reverchonii* leaf	NCED3_ARATH	−2.972	0.005	9-cis-epoxycarotenoid dioxygenase NCED3
	PIP21_ARATH	−3.861	0.001	Aquaporin PIP2–1
	SLAC1_ARATH	−2.648	0.019	Guard cell S-type anion channel SLAC1
*M. reverchonii* root	NCED3_ARATH	3.558	0.008	9-cis-epoxycarotenoid dioxygenase NCED3
	AUX1_ARATH	−2.109	0.053	Auxin transporter protein 1
*M. speciosa* leaf	DRE2A_ARATH	2.209	0.031	Dehydration-responsive element-binding protein 2A
*M. speciosa* root	PYL8_ARATH	−2.532	0.033	Abscisic acid receptor PYL8
	NAC52_ARATH	2.004	0.028	NAC domain containing protein 5

## Discussion

We conducted a comparative transcriptomic study of *Mentzelia* to understand how plants have adapted to and will respond to drought stress across an environmental gradient. The xerophytic *M. filifolia* and semi-arid *M. reverchonii* had a greater response to acute drought shock based on the number of significantly DE transcript-clusters, suggesting that species had adaptive responses in both morphological traits and genetic responses. Using results from the FDR *P*-values, we conclude that the mesophytic *M. speciosa* responded less when experiencing drought shock than the xerophytic and semi-arid adapted species, corresponding with the hypothesis that species from drought-prone environments will mount a greater genetic response than mesophytic species. Although the xerophytic and semi-arid species mounted a greater response than the mesophytic species, they showed opposite patterns in the tissues that responded. The genetic response to drought shock in *M. filifolia* was greater in the leaf than the root tissue, whereas the genetic response in *M. reverchonii* was more apparent in roots than leaves. Each species could have a different reaction time to drought stress, which could have been affected by individual plant size [15]. Plant age affects water and carbon availability, resulting in younger and smaller plants being more susceptible to drought shock [15]. The differences in response in tissues might suggest that the cascade of physiological events within leaves and roots are different between species.

Delayed senescence was differentially expressed in leaves and roots of *M. filifolia* and *M. reverchonii*. Mechanisms associated with delayed senescence, such as jasmonic acid production, auxin regulation, and Serine/threonine-protein kinase CHK1-like protein, were identified among the DE transcript-clusters. Delaying cell death might be a response that has evolved through numerous generations of drought exposure. The historical severity and length of drought experienced by each species could be a factor that determines whether delayed senescence is a utilized pathway. Risking embolism and desiccation in order to delay senescence is an interesting process that should be further studied in xerophytes because it seems to play a major role in their adaptive response drought stress.

The three species in our experiment mounted similar, but not identical, genetic responses when exposed to drought. Many of the selected target genes were not found in our data set, however, because of filtering parameters and the large number of comparisons made during the statistical analysis, many could have been removed from the final dataset. Many target genes experienced greater than two logFC, which is biologically significant and points to common molecular change that occur in response to stress across distantly related species. Although few target genes resulted in significant changes, we determined that genes that play a role in drought tolerance in other plant species are also acting within the plant systems of *Mentzelia*.

The mesophytic *M. speciosa* exhibited a genetic response to drought, but because it is exposed to drought stress less often, it might have responded less quickly to stress, suggesting a less efficient and effective response when compared to species in drier environments. If the response was immediate but subsided quickly, the time of tissue collection could have missed the point with highest differential expression. Alternately, if the mechanisms triggered by drought in *M. speciosa* was delayed, the time of tissue collection could have occurred before the genetic response.

Our sequencing depth was adequate to produce transcriptomes that measured large responses to acute drought-shock. More subtle genetic responses that had lower expression levels were unlikely to have been detected by our sequencing coverage, but could still play an important role in how plants respond to acute drought. Future studies that sequence at greater depths are likely to determine the low expression responses that plants mount as they respond to drought.

### Differentially expressed transcript-clusters in leaves

The greatest response to drought tolerance occurred in the leaves *M. filifolia* (Table 5), which is unsurprising given that leaves are the main site of evapotranspiration and would be the first organs to senesce under drought. The large response of down-regulated transcript-clusters implies that metabolic processes are being down-regulated to conserve water and prevent tissue and cell damage. Transcript-clusters were categorized as transmembrane associated proteins, nucleic acid binding proteins, auxin regulation, rDNA transcription, endonuclease activity, or other enzymatic activities. Four transcript-clusters were identified as proteins involved in stress response, senescence, and wound response. Suppressing drought induced leaf senescence greatly increases drought tolerance in transgenic lines of *Nicotiana tabacum* [55], and *M. filifolia* also appears to be down-regulating senescence-associated proteins. Rivero et al. [55] determined that delaying senescence in transgenic lines of *N. tabacum* increased processes of reactive oxygen species scavenging, leading to extra protection for the photosynthetic apparatus that increased water use efficiency under drought stress. Jasmonic acid, a phytohormone responsible for signal transfer in response to senescence [37], was significantly down-regulated in *M. filifolia*. Down-regulating jasmonic acid, a known senescence accelerator [35], would delay senescence, which appears to be a large component of how *M. filifolia* mitigates drought stress damage in leaves.

Transcription-factor proteins associated with auxin production and transport were down-regulated. Auxin response factors are associated with drought responses because of their role in hormonal response signaling as well as developmental and senescence processes [54]. When exposed to drought stress, developmental processes would most likely stop, inducing down-regulation of auxin. A single auxin related protein was up-regulated in leaves, suggesting a dynamic response in the role of auxin during acute drought stress in *Mentzelia*. Ke et al. [35] showed that when a transgenic line of poplar was designed to overproduce auxin, drought stress tolerance was increased. Auxin metabolism is monitored by many other metabolic pathways and plays a large role in overall plant homeostasis [35]. Similar to the down-regulation of senescence, auxin regulation might be delayed to prevent the occurrence of senescence related processes, but a single occurrence of up-regulation could be attributed to its role as a hormonal response signal or other developmental processes. Overall, *M. filifolia* leaves have a large response of down-regulation involved with senescence brought on by drought stress, and general metabolic processes, like photosynthesis, which would prevent water loss and cell death by attempting to maintain sustainable levels of homeostasis while regulating levels of reactive oxygen species [45].

Two of the seven up-regulated transcript-clusters in *M. filifolia* leaves were endonucleases. Endonucleases act to remove introns by breaking the phosphodiester bonds to produce functional mRNA [74]. The up-regulation of endonucleases might be degrading mRNA [63]. Messenger RNA degradation prevents the translation of proteins involved in metabolic processes that could cause destabilization under stress. Two proteins identified as integral components of the cellular membrane were up-regulated, one specifically identified as TIP1–1 aquaporin protein transmembrane transport, whereas the other is a form of glucosyltransferase involved in the accumulation of the yellow pigment crocetin during fruit development [47]. Aquaporins primarily function to transport water through the cellular membrane, but Zhou et al. [78] determined that the up-regulation of a PIP2 subgroup of aquaporin proteins enhanced drought tolerance in tobacco by increasing the ability to retain water, limit oxidation activity, and decrease the need for antioxidant activity. Aquaporins play an important role in drought stress; however, whether aquaporin proteins are up or down-regulated depends on the species and tissue [59].

*Mentzelia reverchonii* leaves up-regulated two photosynthetic enzymatic processes (Table 7). Ribulose bisphosphate carboxylase/oxygenase (RuBisCO) was up-regulated, suggesting that photosynthesis signaling increased because it cannot function, or it is deactivated when ratios of ATP/ADP become unfavorable due decreased photosynthesis [12]. The metabolic responses typically associated with drought occur as a response to oxidative stress, rather than a direct response to water deprivation [20]. Because *M. reverchonii* has become accustomed to longer periods of drought stress, its initial response to water deprivation might be to increase carbon assimilation to prepare reserves for stress levels that are intolerable by increasing photosynthetic processes instead of immediately shutting down by reducing gas exchange through stomatal closure.

*Mentzelia speciosa* leaves responded to drought stress by up-regulating polyubiquitin-like proteins (Table 9). The most common functional role of ubiquitin is the intracellular control of protein content and degradation [66]*.* Ubiquitin production under stress degrades non-drought stress proteins, which would impair the response to drought stress in an efficient way [66].

### Differentially expressed transcript-clusters in roots

*Mentzelia filifolia* roots up-regulated cellular respiration, while downregulating the production of secondary metabolites and the oxidation-reduction process (Table 6). When photosynthesis is slowed in the above-ground organs of the plant, carbon is allocated from the root’s carbon sinks [26]. Our results based on *M. filifolia*, therefore, suggests that carbon sequestration in roots is vital to mobilize energy when xerophytes experience drought and photosynthesis is shut down. Although Hasibeder et al. [26] determined that root respiration decreased under prolonged drought conditions in *Trisetetum flavescentis*, the initial response of down-regulated photosynthesis might result in higher levels of respiration in roots [20]. Up-regulated respiration suggests that recovery efforts might occur to offset the decreased photosynthetic rate [20].

*Mentzelia reverchonii* roots had a large response to the drought treatment (Table 8). Two of the 54 differentiated transcript-clusters were involved with ubiquitin activity. Increasing the expression of an enzyme responsible for protein degradation would play a direct role in expressed proteins and overall metabolic function [66]. Similar to *M. filifolia* leaves, auxin transport was up-regulated in a single transcript-cluster, suggesting that another function associated with auxin transport was increased, such as hormonal signaling [54].

Transcript-clusters up-regulated in *M. reverchonii* roots were associated with transmembrane transport, specifically sodium-calcium transmembrane transport and cellulose synthase. Calcium transport plays a crucial role in drought, salinity stress signaling, and osmoregulation [29]. Zhu et al. [79] determined that cellulose synthase-like proteins in *Arabidopsis* played a role in osmotic stress tolerance and potentially reactive oxygen species regulation under drought stress. Inositol-tetrakisphosphate regulates the release of intercellular calcium in response to stress [36]. Increasing levels of inositol in *Arabidopsis* and *Solanum* greatly increased drought tolerance and decreased abscisic acid levels [36]. One of the largest transcription factor families, no apical meristem (NAC) experienced up-regulation in *M. reverchonii* roots during drought treatment. NAC transcription factors aid in drought tolerance by the regulation of response pathways [69]. Serine/threonine-protein kinase CHK1-like protein, a signal transducer, transcript cluster showed the largest rate of down-regulation in *M. reverchonii* roots. Although CHK1 kinases involved in the DNA damage response (DDR) system have not been identified in plants, a protein of similar function had a large response to drought stress [75]. Plants trigger a DDR system to regulates cell death and DNA repair under stressful conditions [75]. The *M. reverchonii* response might be delaying the need to utilize the DDR to prevent cell death from occurring, similar to *M. filifolia* leaves delaying senescence.

### Similarities in drought tolerance responses through target gene analysis

Physiological drought responses have a genetic basis [43]. Drought-stress studies on model and crop species have identified common differentially expressed drought-stress genes. Target genes based on previous drought studies were expressed more greatly in *M. filifolia* and *M. reverchonii* than in *M. speciosa*, further supporting the hypothesis that species adapted to drier environments mount a greater response to drought stress.

*Mentzelia filifolia* leaves expressed three target genes: Dehydrin COR47, Protein early responsive to dehydration 15, and E3 ubiquitin-protein ligase SDIR1. E3 ubiquitin-protein ligase SDIR1 acts as a positive regulator of abscisic acid stress signal transduction. Improved drought tolerance has been shown in *Arabidopsis* when over-expression of SIDR1 occurs [76]; however, SDIR1 was under expressed in *Mentzelia*. Dehydrin COR47 produces a dehydrin hydrophilic protein and was over expressed. Dehydrins accumulate in stressed plant tissues associated with dehydration [30]. Dehydration 15 negatively regulates plant response to abscisic acid [34], and down-regulation of abscisic acid decreases drought tolerance in plants [34]. Our results determined that *M. filifolia* is delaying or down-regulating this particular response to drought.

The target genes NAC-56 and PGR5-like protein 1A were expressed in *M. filifolia* roots. NAC-56 in root tissues up-regulate target genes that aid in drought tolerance [10]. No apical meristem transcription factors in transgenic-rice roots enhance drought tolerance by targeting genes responsible for changing root architecture [10]. PGR5-like protein 1A, a thylakoid transmembrane protein, was downregulated. PGR5-like protein 1A plays a direct role in the photosynthetic cyclic electron flow that transport electrons to produce ATP [27]. Downregulating the flow of electron transport would decrease or shut down photosynthesis productivity.

*Mentzelia reverchonii* leaves down-regulated target genes of 9-cis-epoxycarotenoid dioxygenase NCED3, Aquaporin PIP2-1, and Guard cell S-type anion channel SLAC1, while roots up-regulated 9-cis-epoxycarotenoid dioxygenase NCED3 and down-regulated Auxin transporter protein 1 (Table 11), suggesting that the photosynthetic process, or at the very least stomata and transport channels, are being shut down. 9-cis-epoxycarotenoid dioxygenase is a key enzyme in ABA biosynthesis and is induced by drought stress to control the level of endogenous ABA produced [32]. In *M. reverchonii*, ABA is downregulated in leaves, but upregulated in roots. The down-regulation of plasma membrane intrinsic protein aquaporin might be due to it being a low expression aquaporin when constitutively expressed. The down-regulation of a negative regulator of guard cell anion like the R-type channel that responds rapidly to cystolic Ca2+ [58] might be what it is utilized for in *Mentzelia* [64]. An auxin transport protein was down-regulated in the roots of *M. reverchonii*. Down-regulation of molecular and cellular components associated with senescence, like auxin, is regularly down-regulated in *Mentzelia*, and might be a driving factor in how multiple species tolerate drought stress.

Three target genes were DE in *M. speciosa* leaves and roots (Table 11). Leaves overexpressed Dehydration-responsive element-binding protein 2A, which is a gene element that helps regulate expression of genes utilized to cope with drought [52]. Over expression within leaves might be the first response of the cascade of mechanisms plants use to avoid damage from drought stress. A single target gene, NAC domain containing protein 52, was up-regulated in *M. speciosa*. Similar to *M. filifolia*, NAC-56 in roots is a NAC transcription factor that up-regulates a group of target genes that aid in drought tolerance [10], and the up-regulation of NAC-56 might be to change root architecture to adapt to drought stress [10]. The gene probable pectate lyase 8 (PLY8) was down-regulated within *M. speciosa* roots. PLY8 is necessary for lateral root growth after inhibition by abscisic acid [77]. Down-regulation is probably attributed to inhibition through ABA, which is produced in response to drought stress in roots.

### Tissue response between species

Leaves and roots responded differently to drought shock depending on species. When leaves were compared among species, the xeric *M. filifolia* and semi-arid *M. reverchonii* mounted a stronger response than the mesic *M. speciosa* (Fig. 4). In contrast, when comparing responses in roots, the xerophytic *M. filifolia* mounted the weakest response compared to the other two species. The genetic response, consequently, appears to be organ and environmental dependent. Although *M. speciosa* mounted a greater response to drought in roots compared to *M. filifolia*, overall *M. speciosa* responded less to drought shock compared to both *M. reverchonii* and *M. filifolia*.

The differences in tissue response suggests that drought response is tissue, species, and environment specific, and selection pressures related to drought response might be acting on tissues differently. Further research is needed to determine if selection is acting on leaf and root tissues separately. The cascade of physiological response and water regulation described by Bartlett et al. [4] could be tissue specific and instead of studying response as a function of the whole plant, future research should focus on specific tissue responses. Manipulation of genes that play a role in roots or leaves independently might lead to novel pathways for genetic modification allowing for greater drought tolerance that is not only tissue specific but serves to enhance drought tolerance overall.

## Conclusions

Roots and leaves respond to acute drought through different pathways, which are influenced by the environments in which they evolve. Differential expression of genes in response to drought in roots or leaves might lead to novel pathways for genetic modification, allowing for greater drought tolerance that is not only tissue specific, but serves to enhance drought tolerance overall. Delayed senescence played a much larger role than anticipated in both leaves and roots of *M. filifolia* and *M. reverchonii*. The ability to delay cell death might be a response that has evolved through generations of drought exposure. Risking embolism and desiccation in order to delay senescence is an curious process that should be further studied in xerophytes because it seems to play a major role in their adaptation to drought stress. *Mentzelia speciosa* produced the weakest response to drought despite having the broadest leaves and occurring in the most mesophytic environment. The individuals of *M. speciosa* had been exposed to less frequent occurrences of drought, potentially leaving them without the evolved ability to respond in an efficient and timely way. We also observed differences in drought response depending on tissue type suggesting that species could have a mounted response within one tissue type and not the other, and tissue-specific responses could be evolving at different rates. Further studies that utilize quantitative qPCR would help to verify the presence of target genes within tissues and the magnitude of the regulation taking place. Our results suggest that, in additional to morphological evolution to limit drought stress, xerophytes have evolved a cascade of genetic responses that have tissue-specific responses to mitigate drought stress through delayed senescence, decreased photosynthesis, and decreased water transport.

## Methods

**Fig. 6:**
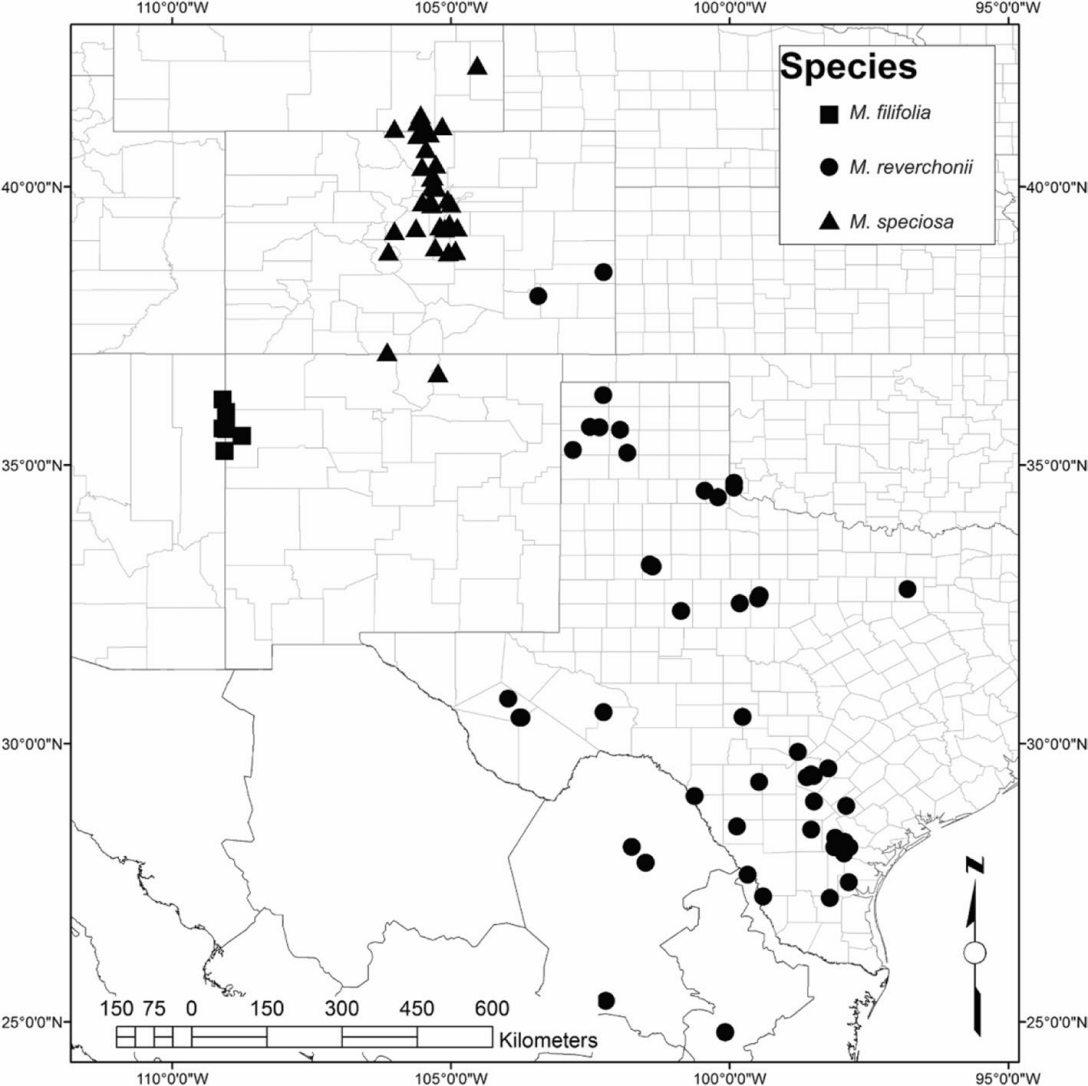
Distribution of *Mentzelia* species in western North America that were used in this study

Species of *Mentzelia* occur across a wide environmental gradient, from southwestern North American deserts, to mesic habitats near the Continental Divide in the Rocky Mountains [60]. Despite their ecological importance across western North America, and especially in drought-prone gypsum outcrops [60, 61], we do not understand the mechanisms behind their drought tolerance and success in xeric habitats. Three species of *Mentzelia* were sampled that occur across an environmental gradient, from desert to mesic ecosystems (Fig. 6). The xerophytic *M. filifolia* was sampled in the New Mexican Chihuahuan Desert. *Mentzelia reverchonii*, a semi-arid species, was collected in the Texas short-grass prairies. The mesophytic *M. speciosa* was sampled in the central Rocky Mountains of Colorado. Natural populations of all three species were sampled because plants failed to grow in greenhouse conditions. The three sampled species belong to the same section within *Mentzelia*, section *Bartonia*, but are not each other’s closest relatives [62]. At the time of collection, the *M. filifolia* population in New Mexico received 2.16 cm of precipitation during July and has a mean annual precipitation of 19.71 cm [16]. The *M. reverchonii* population in Texas received 4.24 cm of precipitation for June, with a mean annual precipitation of 53.19 cm [16]. The *M. speciosa* population in Colorado received 3.3 cm of precipitation in July, and has a mean annual precipitation of 55.60 cm [16]. All habitats experienced average rates of rainfall for July according to precipitation data from the past 30 years.

### Field sampling

Natural populations were sampled on separate dates in the months of June and July, 2017. *Mentzelia filifolia* was collected northwest of Gallup, New Mexico on July 12th, 2017 (35.65186°N, 109.02622°W, 2080 m). Sampling occurred at 5:00 PM with a temperature of 31 °C. Individuals of *M. reverchonii* were collected in Shackelford County, near Fort Phantom Lake in Abilene, Texas on June 27th, 2017 (32.606747°N, 99.692199°W, 511 m). Sampling occurred at 6:00 PM with a temperature of 32 °C. Individuals of *M. speciosa* were collected southwest of Lyons, Colorado on July 5th, 2017 (40. 202,972°N -105.299625°W, 1900 m). Sampling occurred at 2:00 PM with a temperature of 32 °C. Four control and four treatment plants that were developmentally identical (bolted from the rosette stage with flowers present) were selected randomly while maximizing distance between them to avoid sampling closely related individuals. Four treatment plants were excavated with their roots intact and placed on the ground in full sun. We refer to this approach as a drought-shock treatment, which has been used in other studies to examine the response to drought in natural populations (e.g., [48, 56]). While the drought-shock treatment was being conducted, four control plants were excavated with roots intact and sampled immediately to avoid sampling drought-stressed tissues. Control plants were excavated prior to leaf sampling to ensure that any wound response associated with the extraction from the ground would be identified in both the control and treatment plants, which would result in no differentially expressed (DE) genes associated with wounding after applying our bioinformatics pipeline (see below). Leaf and root tissues were collected in replicates of three, while flower, fruit, and stem tissues were collected from one individual per population for the purpose of generating a reference transcriptome. Mature leaf tissues with no insect damage were immediately placed in 1.5 mL of RNA*later*™ RNA Stabilization Solution (Thermo Fisher Scientific, Waltham, Massachusetts, U.S.A.) to preserve the RNA. Root tissues were sampled after the leaf tissues were sampled. Excess soil was removed by brushing off the roots, but much of the remaining soil washed away before RNA extraction. Approximately 100 g of tissue from the middle of the central tap root was sampled. The treatment plants were subjected to an hour of full sun exposure until leaves began to wilt and curl, indicating that the plants were experiencing acute drought stress. All tissue samples were placed in a − 80 °C freezer approximately 3–5 days after collection until the RNA was extracted. Vouchers for the collected population were pressed and deposited in the Georgia Southern University Herbarium (GAS).

### RNA extraction

Tissue samples were thawed, removed from RNA*later*, and placed into new tubes with 2.8 mm ceramic beads. Samples were frozen and homogenized with a Qiagen Tissuelyser II (Qiagen, Hilden, Germany) for 1 min at 30 Hz. TRIzol extraction buffer (Thermo Fisher Scientific) was added in aliquots of 1 mL to each sample, homogenized for nine additional minutes, and then incubated for 5 min at room temperature. The phase separator, 1-bromo-3-chloropropane, was added in 100 μl aliquots, vortexed for 15 s, and incubated at room temperature for 5 min. Samples were centrifuged at 12,000 g for 10 min. The upper aqueous phase was transferred to a new tube and 500 μl of chilled isopropanol was added. The samples were stored overnight in a − 80 °C freezer. After approximately 14 h, the RNA samples were removed from the freezer and centrifuged at 12,000 g for 10 min. The supernatant was discarded, 1 mL of chilled 75% EtOH was added, and samples were centrifuged for 5 min at 12,000 g. All supernatant was removed, and samples were air dried in a fume hood for 30 min. The RNA was re-suspended into 60 μl of nuclease-free water. The concentration of each RNA sample was quantified using both a Qubit Fluorometer (Qubit 2.0; Invitrogen, Life Technologies, California, U.S.A.) and an Agilent 2100 Bioanalyzer (Agilent Technologies, Inc., Waldbronn, Germany).

### cDNA library creation

cDNA libraries were created from each RNA isolation. We used the NEBNext Ultra RNA Library Prep Kit for Illumina (New England Biolabs, Ipswich, Massachusetts, U.S.A.) in conjunction with the NEBNext Poly(A) mRNA Magnetic Isolation Module (New England Biolabs) and the NEBNext Multiplex Oligos for Illumina (New England Biolabs). All libraries were generated following the manufacturer’s protocol. Both the Qubit Fluorometer (Qubit 2.0 HS DNA assay) and the Agilent 2100 Bioanalyzer were used to quantify each library. All cDNA libraries were pooled together to maintain a 10 mM concentration, and then sequenced on an Illumina NextSeq (150 Cycles) PE75 High Output flow cell at the Georgia Genomics and Bioinformatics Core at the University of Georgia.

### Reference transcriptome generation, annotation, and comparisons

Because no reference transcriptome is published for *Mentzelia*, we generated de novo transcriptome assemblies that were applied as references in subsequent analyses. Raw-sequence read-quality was assessed with the FASTX-Toolkit [22]. Reads were quality filtered and trimmed in Trimmomatic v0.36 [7] to remove adapter sequences, ambiguous nucleotides, low quality sequences with Phred scores ≤20, and sequences < 36 bp in length [42]. Flower, fruit, stressed and unstressed roots, stressed and unstressed leaves, and stem tissue sequences were combined to generate two separate reference transcriptomes of *M. speciosa* and *M. filifolia*. Sequence reads were assembled in Trinity v2.4.0 [25] for de novo generation [23] with a K-mer size of 25, which is a sufficient size for a de novo assembly for a non-model organism with no reference genome [24].

To ensure that each transcriptome was complete and an adequate representation of both species, HISAT2 v2.0.5 [51] was used to map back the trimmed sequence reads from each sample. Average alignment percentages were calculated to ensure at least an 80% alignment rate average.

Trinotate (https://trinotate.github.io/) was used for the comprehensive de novo transcriptome annotation. Trinotate utilizes BLAST [1] and SwissProt [6] to infer homology based on sequence similarity, HMMER [19] and PFAM [3] for protein domain identification, and eggNOG [31], GO [2, 8, 21], and KEGG databases [33] to identify functional groups or pathways. All programs were used in conjunction with Trinotate to create a functional annotation for each transcriptome generated using the output from each search to populate an SQlite database [70]. The SQlite database was used to create an annotation report showing all results from each respective database search.

HISAT2 [51] was used to assess the completeness and quality of the transcriptome alignments by re-mapping the trimmed reads back to the transcriptome alignment. A BUSCO [67] analysis was performed on the remapped reads to determine the completeness of each de novo assembled transcriptome by comparing present single-copy orthologs using the provided eukaryotic lineage dataset to the generated annotated assembly.

### Expression analysis

Burrows-Wheeler Aligner (BWA) v0.7.13 [39] determined transcript level abundance by mapping low divergence transcript sequences to a reference transcriptome. We employed the *M. filifolia* and *M. speciosa* reference transcriptomes to conduct reference-guided assemblies. Reference transcriptomes for the respective species were used as the target inputs, using the reference from the closely related *M. speciosa* for *M. reverchonii* [62]. Each transcriptome was made into an FM-index to compress full text files for faster alignment rates. After the indices were made, the options for a mismatch penalty of 0.05, a gap open penalty of one, no output lower than 10, 20 threads, and “mem” option for local alignment of transcripts back to the reference were used for BWA analysis. SAMtools v1.3 [40] was used to convert the SAM output files from BWA to sorted BAM files, which were inputted into Corset v1.07 [13] that hierarchically grouped transcript contigs into clusters by shared reads and expression data. Counts of the number of transcripts included in each cluster were made and used as the input raw count data for differential expression analyses. The edgeR package [44, 57] was used for differential expression analysis in R [53]. Transcript-cluster count files were read in by species and tissue type separately (e.g., root tissues of *M. speciosa*), with individuals grouped together by control or treatment. The DGEList function was used to create an object from the transcript cluster-counts for each species and tissue type individually. Transcript clusters with fewer than one transcript count per million in fewer than six of the eight individuals were discarded to reduce the number of rarely expressed genes that were not DE across all members of a group. Normalized factors were calculated to scale each library size. Common dispersion was calculated to maximize the negative binomial, conditional common-likelihood to estimate a common dispersion value across all genes. Tagwise dispersion was estimated with an empirical Bayes method based on weighted conditional maximum likelihood [44, 57]. We used the exact test to determine differences in mean values between the two negative binomially distributed counts. A false discovery rate *P*-value adjustment was used to address multiple comparisons. We also compared DE clusters with log-fold changes of two or greater. Differentially expressed clusters were annotated with Blast2Go [11] to identify their gene or protein name, along with a description and function.

Bivariate plots were generated in R, and differentially expressed genes with a log-fold values of 2 or greater were indicated. Differential expression values were estimated by comparing the treatment to the control for each species. A slope of one is expected if expression levels between species are identical, and we tested whether expression significantly deviated with the one-sample slope-test with the smatr package [71] in R. Statistical tests were conducted by constructing distributions for the test statistic following Taskinen and Warton [68].

### Target gene approach

Complementary to the transcriptome profiling approach, we applied a target gene approach to search for expression patterns in drought associated genes. In comparison to approaches that strictly profile the transcriptome for significantly DE genes, a targeted approach has the ability to determine the exact levels of differential expression [43] and whether genes associated with drought response are expressed at all. The target approach can determine commonalities in stress response across plants, allowing us to identify genes that commonly or uniquely respond to drought. We took advantage of previous studies to create a list of drought-tolerant genes and their sequences, then explicitly measured the response of the targeted genes to determine if *Mentzelia* responded similarly to drought stress.

We selected 90 genes from different gene families known to play a role in drought response (e.g., [9, 35, 38, 41, 46, 49, 50, 65, 78]), as well as genes with GO terms related to or associated with drought tolerance from GenBank [5]. The target genes from *Arabidopsis*, corn, soy bean, and sorghum were downloaded from GenBank. The entry name for each target gene was searched within sequence annotation reports from the reference transcriptomes to identify the associated transcript-cluster’s ID. The edgeR results from the differential expression exact test for each species and tissue type was searched using the annotated genes or protein names to identify if the associated gene clusters and the level of gene expression for each target gene.

## Supplementary Information

**Additional file 1 **: **Supplemental Data.** Log-fold change in expression levels of target genes found in the results of the exact test to determine differential expression for each tissue type, root and leaf, and all three species; *Mentzelia filifolia*, *M. reverchonii*, and *M. speciosa*. Values for log-fold changes (LogFC) show the direction and extent of the change of expression for each target gene. Values of LogFC with * indicate values with corresponding significant *p*-values <0.05.

## Data Availability

The datasets generated and/or analyzed during the current study are available in the NCBI GenBank repository: ncbi.nlm.nih.gov/genbank/. Raw sequence reads and assembled transcriptome data have been deposited under the BioProject accession number: PRJNA708198.

## Abbreviations

ABA: Abscisic acid
ATP: Denosine triphosphate
DE: Differential expression
DDR: DNA damage response
FDR: False discovery rate
HA-1: Alternative hypothesis 1
HA-2: Alternative hypothesis 2
HA-3: Alternative hypothesis 3
Ho: Null hypothesis
logFC: Log-fold change
mRNA: Messenger RNA
NAC: No apical meristem
rDNA: Ribosomal DNA
RuBisCO: Ribulose bisphosphate carboxylase/oxygenase
