# Supplementary material for: Inferring the genetic responses to acute drought stress across an ecological gradient
Source: BMC Genomics. 2022 Jan 4;23:3. doi: 10.1186/s12864-021-08178-w (PMC8725310; doi:10.1186/s12864-021-08178-w)
Supplement: Supplementary file 1 — Additional file 1 : Supplemental Data. Log-fold change in expression levels of target genes found in the results of the exact test to determine differential expression for each tissue type, root and leaf, and all three species; Mentzelia filifolia, M. reverchonii, and M. speciosa. Values for log-fold changes (LogFC) show the direction and extent of the change of expression for each target gene. Values of LogFC with * indicate values with corresponding significant p-values <0.05. [file 12864_2021_8178_MOESM1_ESM.pdf]

Supplemental Data: Log-fold change in expression levels of target genes found in the results of the exact test to determine differential expression for each tissue type, root and leaf, and all three species; *Mentzelia filifolia*, *M. reverchonii*, and *M. speciosa*. Values for log-fold changes (LogFC) show the direction and extent of the change of expression for each target gene. Values of LogFC with * indicate values with corresponding significant *p*-values <0.05.

Target Gene	*M. filifolia* leaf	LogFC	*M. filifolia* root	LogFC	*M. reverchonii* leaf	LogFC	*M. reverchonii* root	LogFC	M. *speciosa* leaf	LogFC	*M. speciosa* root	*LogFC*
14333_ORYSJ	NF	—	NF	—	NF	—	NF	—	NF	—	NF	—
PSD8A_ARATH	22463.0	—	66259.0	—	12249.0	—	47360.0	—	18856.0	—	64380.0	1.36
—	8305.0	—	—	—	51036.0	-1.24	51649.0	0.62	31880.0	0.32	66290.0	—
—	27506.0	—	—	—	66230.0	—	49806.0	—	8982.0	—	74369.0	—
—	—	—	—	—	26428.0	-0.02	18766.0	—	23097.0	-0.04	73950.0	0.38
—	—	—	—	—	28677.0	*-1.40	44515.0	—	38364.0	0.56	75718.0	-0.08
KCS12_ARATH	59862.0	—	NF	—	35325.0	-0.25	31896.0	—	30958.0	-0.39	NF	—
NCED3_ARATH	37429.0	—	54725.0	—	78234.0	*-2.97	48884.0	*3.56	40341.0	-1.47	79148.0	0.66
ABAH3_ARATH	NF	—	NF	—	NF	—	NF	—	NF	—	NF	—
PYL4_ARATH	NF	—	64731.0	—	36918.0	—	15804.0	—	14619.0	—	40971.0	—
—	—	—	—	—	38747.0	—	21971.0	—	19339.0	0.63	42598.0	—
—	—	—	—	—	43509.0	—	24931.0	-1.22	38723.0	0.39	80026.0	—
—	—	—	—	—	46098.0	—	26662.0	—	—	—	—	—
—	—	—	—	—	51985.0	—	—	—	—	—	—	—
PYL5_ARATH	NF	—	NF	—	32554.0	—	7019.0	—	16691.0	—	62278.0	—
PYL8_ARATH	15351.0	—	65443.0	0.26	52581.0	-1.07	36519.0	—	21321.0	-0.79	56716.0	—
—	51713.0	—	30869.0	—	56051.0	—	40593.0	—	33428.0	—	65307.0	—
—	9070.0	—	31615.0	—	56561.0	—	53179.0	—	46297.0	—	68408.0	*-2.53
—	22672.0	0.33	—	—	69954.0	—	55647.0	—	12507.0	—	74099.0	—
—	60472.0	1.19	—	—	79678.0	—	49611.0	—	43435.0	0.29	77705.0	—
—	—	—	—	—	9053.0	—	28605.0	1.26	26244.0	-0.06	86165.0	—
—	—	—	—	—	51531.0	-0.38	54711.0	—	32619.0	0.71	48696.0	-0.88
—	—	—	—	—	56894.0	0.45	680.0	—	—	—	61932.0	-1.01

—	—	—	—	—	61592.0	—	—	—	—	—	43429.0	—
AI5L5_ARATH	1997.0	—	20640.0	0.72	14599.0	—	15949.0	—	16488.0	—	58616.0	0.18
—	31329.0	0.40	27826.0	—	17403.0	-0.21	21743.0	-0.28	32826.0	—	60111.0	—
—	32392.0	—	59294.0	0.64	36195.0	—	38777.0	—	33412.0	-0.03	79315.0	—
—	60308.0	-1.29	63349.0	—	55221.0	—	39414.0	—	52576.0	-1.79	79821.0	—
—	—	—	—	—	73585.0	—	42416.0	—	35463.0	—	80601.0	0.32
—	—	—	—	—	79680.0	-0.44	47950.0	—	—	—	85753.0	—
—	—	—	—	—	14790.0	0.80	52731.0	—	—	—	71374.0	—
—	—	—	—	—	—	—	55548.0	—	—	—	—	—
—	—	—	—	—	—	—	33036.0	—	—	—	—	—
AGAL_ORYSJ	NF	—	NF	—	NF	—	NF	—	NF	—	NF	—
Q39958_HELAN	NF	—	NF	—	NF	—	NF	—	NF	—	NF	—
PIP21_ARATH	NF	—	NF	—	51261.0	*-3.86	21024.0	—	16268.0	—	66614.0	—
—	—	—	—	—	2192.0	—	—	—	38156.0	—	62374.0	—
—	—	—	—	—	13245.0	—	—	—	—	—	—	—
—	—	—	—	—	22082.0	—	—	—	—	—	—	—
—	—	—	—	—	24320.0	—	—	—	—	—	—	—
—	—	—	—	—	4169.0	—	—	—	—	—	—	—
—	—	—	—	—	17427.0	—	—	—	—	—	—	—
—	—	—	—	—	75869.0	—	—	—	—	—	—	—
—	—	—	—	—	9599.0	—	—	—	—	—	—	—
—	—	—	—	—	57346.0	—	—	—	—	—	—	—
—	—	—	—	—	61247.0	1.33	—	—	—	—	—	—
—	—	—	—	—	71024.0	—	—	—	—	—	—	—
SPE1_ARATH	NF	—	NF	—	NF	—	NF	—	NF	—	NF	—
RQL1_ARATH	27256.0	—	52877.0	—	1732.0	—	41198.0	—	29034.0	—	81069.0	—
—	26364.0	—	28967.0	—	60935.0	—	—	—	—	—	—	—
—	—	—	31648.0	—	79488.0	—	—	—	—	—	—	—
RQL5_ARATH	NF	—	NF	—	15530.0	—	NF	—	37859.0	—	55195.0	—
—	—	—	—	—	50086.0	—	—	—	—	—	83738.0	—
—	—	—	—	—	79234.0	—	—	—	—	—	—	—
SUV3M_ORYSJ	34806.0	—	NF	—	NF	—	NF	—	NF	—	NF	—
ARFG_ARATH	NF	—	NF	—	NF	—	NF	—	NF	—	NF	—
BIG_ARATH	20176.0	—	27359.0	—	21102.0	—	19408.0	—	30289.0	-0.60	34102.0	—
—	21773.0	-0.24	28837.0	0.63	21538.0	—	29644.0	—	40436.0	-0.01	48303.0	*1.71
—	48276.0	0.84	33816.0	0.33	75461.0	-1.19	13916.0	—	7679.0	—	53644.0	*1.91
—	57508.0	—	33972.0	0.11	32151.0	—	—	—	—	—	28180.0	—
—	57508.1	—	49773.0	—	—	—	—	—	—	—	—	—
—	59018.0	—	58797.0	0.53	—	—	—	—	—	—	—	—

—	40523.0	*-1.53	63926.0	—	—	—	—	—	—	—	—	—
—	32488.0	-0.44	65540.0	—	—	—	—	—	—	—	—	—
—	—	—	66590.0	—	—	—	—	—	—	—	—	—
AUX1_ARATH	41000.0	0.13	48388.0	—	22458.0	-0.44	19406.0	-1.19	19013.0	1.09	49241.0	—
—	56453.0	-1.08	59761.0	1.89	23034.0	—	24315.0	—	24409.0	—	67389.0	0.20
—	—	—	67537.0	1.47	42284.0	—	44098.0	-2.11	38423.0	—	73976.0	—
—	—	—	—	—	59703.0	*-1.69	46672.0	—	43511.0	—	81710.0	—
—	—	—	—	—	60652.0	-0.42	57621.0	—	52709.0	—	91781.0	-0.19
—	—	—	—	—	51426.0	—	14507.0	—	—	—	60027.0	0.54
—	—	—	—	—	—	—	12132.0	—	—	—	—	—
Q6IVL3_GOSHI	NF	—	NF	—	NF	—	NF	—	NF	—	NF	—
CDPKN_ARATH	NF	—	NF	—	NF	—	NF	—	NF	—	NF	—
CB22_ARATH	NF	—	NF	—	NF	—	NF	—	NF	—	NF	—
CB3_ARATH	33647.0	0.88	29531.0	—	28158.0	—	11689.0	—	21075.0	—	50686.0	—
CA4_ARATH	40546.0	*-1.89	12301.0	—	43886.0	—	NF	—	NF	—	8266.0	—
—	40113.0	*-1.70	20719.0	—	9244.0	—	—	—	—	—	42550.0	—
—	28496.0	—	—	—	63696.0	1.01	—	—	—	—	45962.0	—
—	—	—	—	—	13901.0	—	—	—	—	—	47470.0	—
—	—	—	—	—	—	—	—	—	—	—	60006.0	0.53
—	—	—	—	—	—	—	—	—	—	—	88113.0	0.04
CSP2_ARATH	NF	—	NF	—	NF	—	NF	—	NF	—	NF	—
DRE1A_ARATH	20891.0	—	69584.0	—	NF	—	NF	—	NF	—	NF	—
DRE1B_ARATH	31625.0	—	31046.0	—	NF	—	NF	—	NF	—	NF	—
—	—	—	39486.0	—	—	—	—	—	—	—	—	—
—	3975.0	—	46882.0	—	—	—	—	—	—	—	—	—
—	—	—	46882.0	—	—	—	—	—	—	—	—	—
DRE1C_ARATH	NF	—	NF	—	49962.0	—	2184.0	—	26626.0	—	35024.0	—
—	—	—	—	—	—	—	4186.0	—	40833.0	-1.07	48587.0	1.09
DRE2A_ARATH	NF	—	32555.0	—	57685.0	—	37216.0	—	53037.0	—	65485.0	—
—	—	—	48161.0	—	68844.0	—	1348.0	—	40862.0	*2.21	51543.0	—
—	—	—	—	—	36498.0	—	2488.0	—	53836.0	*1.99	90226.0	-0.41
COR47_ARATH	42834.0	*2.10	27118.0	0.87	3433.0	—	NF	—	45038.0	—	49053.0	—
—	—	—	60204.0	—	NF	—	—	—	—	—	—	—
RZF1_ARATH	27763.1	—	35403.0	—	19813.0	—	NF	—	NF	—	NF	—
SDIR1_ARATH	56824.0	*-1.96	35045.0	-0.27	20889.0	—	55882.0	—	30086.0	—	43117.0	—
—	20382.0	—	44788.0	0.39	60311.0	—	1710.0	—	33144.0	—	65865.0	—
—	43020.0	-0.84	48693.0	—	66070.0	—	35587.0	—	10214.0	—	31466.0	—
—	—	—	—	—	18538.0	—	29054.0	—	—	—	29667.0	—
A0A1S2Z179_CICAR	NF	—	NF	—	NF	—	NF	—	NF	—	NF	—

SINA3_ARATH	51265.0	-0.26	26018.0	—	33582.0	0.41	42684.0	-0.52	43359.0	0.19	87037.0	-0.73
—	32570.0	-0.01	34484.0	—	18831.0	-0.58	46118.0	0.69	43690.0	-0.17	77433.0	-0.84
—	47711.0	-1.47	53934.0	0.34	—	—	—	—	—	—	—	—
—	—	—	60529.0	0.55	—	—	—	—	—	—	—	—
ELP1_ARATH	39468.0	-0.21	29575.0	0.43	51648.0	—	52021.0	—	Cluster-20592.0	—	44084.0	—
—	9730.0	—	—	—	42238.1	0.17	52021.2	-0.40	Cluster-43395.0	-0.13	82243.0	-1.38
—	35892.0	-0.10	—	—	42238.0	—	52021.1	—	—	—	38666.0	—
—	62044.0	-0.36	—	—	—	—	—	—	—	—	—	—
ABI4_ARATH	NF	—	NF	—	NF	—	NF	—	NF	—	NF	—
WIN1_ARATH	NF	—	45954.0	—	28899.0	—	1632.0	—	50935.0	—	NF	—
—	—	—	—	—	3476.0	—	19788.0	—	25222.0	—	—	—
ALFC7_ARATH	NF	—	NF	—	NF	—	NF	—	NF	—	NF	—
GOLS3_ARATH	NF	—	NF	—	NF	—	NF	—	NF	—	NF	—
GSTFA_ARATH	NF	—	41497.0	—	NF	—	NF	—	NF	—	NF	—
GSTUJ_ARATH	NF	—	NF	—	NF	—	NF	—	NF	—	NF	—
HPR1_ARATH	17372.0	—	36501.0	—	73490.0	—	364.0	—	9949.0	—	86714.0	—
—	60671.0	—	57114.0	—	56851.0	—	—	—	35920.0	—	—	—
GLPK_ARATH	NF	—	NF	—	NF	—	NF	—	NF	—	48301.0	—
GLYR1_ARATH	35836.0	—	37301.0	—	63668.0	—	57557.0	—	39568.0	-0.67	81750.0	—
—	—	—	—	—	1141.0	—	31059.0	—	8548.0	—	56776.0	—
—	—	—	—	—	13446.0	—	—	—	13094.0	—	5765.0	—
—	—	—	—	—	46780.0	0.01	—	—	—	—	64278.0	—
—	—	—	—	—	52601.0	—	—	—	—	—	—	—
SLAC1_ARATH	7432.0	—	NF	—	63580.0	*-2.65	25632.0	—	35818.0	0.87	NF	—
AHK1_ARATH	28013.0	—	41916.0	—	24650.0	—	18000.0	—	46685.0	—	77788.0	—
—	63284.0	-0.20	4422.0	—	49294.1	—	40232.0	—	19410.0	0.04	81623.0	—
—	—	—	—	—	24650.1	-0.98	—	—	—	—	6718.0	—
AHK3_ARATH	NF	—	NF	—	NF	—	NF	—	NF	—	NF	—
AHK4_ARATH	37288.0	1.64	19175.0	—	59014.0	1.17	32554.0	-1.41	23810.0	-0.56	74833.0	-0.59
—	40197.0	—	41092.0	1.63	—	—	—	—	—	—	—	—
—	56831.0	1.46	44187.0	1.74	—	—	—	—	—	—	—	—
—	39205.0	—	—	—	—	—	—	—	—	—	—	—
AHP5_ARATH	33135.0	0.44	44232.0	0.31	3642.0	—	NF	—	16727.0	—	42384.0	—
—	30617.0	0.06	67132.0	-0.79	—	—	—	—	—	—	—	—
YUC6_ARATH	NF	—	NF	—	45456.0	—	41005.0	—	50129.0	—	22995.0	—
—	—	—	—	—	8311.0	—	—	—	9948.0	—	—	—
—	—	—	—	—	54283.0	—	—	—	—	—	—	—

B6UH99_MAIZE	NF	—	NF	—	NF	—	NF	—	NF	—	NF	—
Q9M0X3_ARATH	NF	—	NF	—	NF	—	NF	—	NF	—	NF	—
F4JQF1_ARATH	NF	—	NF	—	NF	—	NF	—	NF	—	NF	—
LEA46_ARATH	6646.0	—	11690.0	—	11294.0	—	NF	—	NF	—	78366.0	—
—	6725.0	—	52317.0	1.09	—	—	—	—	—	—	—	—
—	32615.0	—	10766.0	—	—	—	—	—	—	—	—	—
LEA41_ARATH	NF	—	NF	—	NF	—	NF	—	NF	—	NF	—
LACS2_ARATH	37578.0	0.74	44475.0	—	74518.0	—	9880.0	—	23105.0	—	NF	—
—	—	—	—	—	63173.0	—	—	—	—	—	—	—
MED25_ARATH	40951.0	-1.21	62487.0	0.49	40206.0	—	37187.0	—	38565.0	—	29020.0	—
VDAC3_ARATH	NF	—	NF	—	NF	—	NF	—	NF	—	NF	—
PUMP1_ARATH	62786.0	—	48804.0	—	33421.0	—	22483.0	—	13619.0	—	57109.0	—
—	8716.0	—	55602.0	—	54638.0	—	36943.0	—	30795.0	0.35	46907.0	—
—	43319.0	0.28	65832.0	-0.04	27543.0	-0.13	41493.0	1.18	—	—	48231.1	0.27
M2K1_ARATH	NF	—	NF	—	NF	—	NF	—	NF	—	NF	—
MP3K2_ARATH	NF	—	NF	—	NF	—	NF	—	NF	—	NF	—
NAC52_ARATH	NF	—	NF	—	52816.0	-0.10	52054.0	—	35797.0	0.78	33176.0	*2.00
—	—	—	—	—	—	—	559.0	—	46290.0	0.97	80639.0	—
NAC40_ARATH	37788.0	—	NF	—	23175.0	-0.58	14773.0	—	43811.0	-1.12	86643.0	1.00
—	—	—	—	—	—	—	43581.0	0.82	—	—	—	—
NAC48_ORYSJ	56568.0	0.42	32595.0	—	NF	—	NF	—	NF	—	NF	—
NAC53_ARATH	27987.0	-1.47	53278.0	0.20	43602.0	—	25392.0	—	24549.0	-0.88	67740.0	—
—	—	—	—	—	75638.0	-1.12	46806.0	0.06	47866.0	-0.03	70886.0	-0.14
NAC62_ARATH	NF	—	NF	—	NF	—	NF	—	NF	—	NF	—
NAC56_ARATH	28107.0	—	48011.0	*2.90	18762.0	—	29455.0	—	15242.0	—	56652.0	—
—	51001.0	0.00	—	—	48643.0	—	49666.0	—	50239.0	—	—	—
—	—	—	—	—	57201.0	—	—	—	—	—	—	—
NDUS1_ARATH	NF	—	NF	—	NF	—	NF	—	NF	—	NF	—
AKRC9_ARATH	10817.0	—	29343.0	-0.22	2082.0	—	23710.0	—	44077.0	—	37016.0	—
—	44870.0	0.68	39628.0	—	46868.0	—	52521.0	—	28509.0	0.71	85265.0	—
—	—	—	—	—	63167.0	-0.11	37228.0	—	—	—	87849.0	—
—	—	—	—	—	—	—	—	—	—	—	69958.0	1.53
NPC4_ARATH	NF	—	NF	—	73777.0	—	35738.0	1.57	14807.0	—	66947.0	0.00
—	—	—	—	—	63382.0	—	49197.0	—	16543.0	—	87126.0	—
Q5U9M2_ORYSJ	NF	—	NF	—	NF	—	NF	—	NF	—	NF	—
Q0J265_ORYSJ	NF	—	NF	—	NF	—	NF	—	NF	—	NF	—
PER33_ARATH	NF	—	NF	—	NF	—	NF	—	NF	—	NF	—
PER34_ARATH	NF	—	NF	—	NF	—	NF	—	NF	—	NF	—
PGL1A_ARATH	41556.0	—	34846.0	*-3.47	14163.0	0.72	54107.0	—	37867.0	0.64	88780.0	—

—	45791.0	0.46	47604.0	—	—	—	—	—	—	—	—	—
RFS5_ARATH	33272.0	1.14	29161.0	-1.07	76965.0	*-1.81	42554.0	—	31224.0	0.25	24183.0	—
ABI5_ARATH	NF	—	NF	—	NF	—	NF	—	NF	—	NF	—
AB1K1_ARATH	NF	—	NF	—	18052.0	0.63	18851.0	—	21916.0	0.59	85765.0	—
DTX43_ARATH	38777.0	-0.51	31816.0	1.31	74815.0	—	53199.0	—	NF	—	35781.0	0.36
ERD15_ARATH	51610.0	—	56471.0	0.83	49900.0	—	11294.0	—	12794.0	—	NF	—
—	45143.0	*2.03	23659.0	—	53720.0	—	21884.0	—	—	—	—	—
—	—	—	—	—	54119.0	—	33027.0	—	—	—	—	—
MET1_ARATH	11490.0	—	35467.0	—	53131.0	—	3381.0	—	42413.0	—	52721.0	—
—	4644.0	—	47231.0	—	67555.0	—	1241.0	—	8864.0	—	64336.0	—
—	59675.0	1.68	62256.0	—	77088.0	—	—	—	—	—	—	—
—	—	—	62817.0	—	—	—	—	—	—	—	—	—
Q7XJ04_ORYSJ	NF	—	NF	—	NF	—	NF	—	NF	—	NF	—
PPOX1_ARATH	NF	—	NF	—	3876.0	—	NF	—	NF	—	61957.0	—
KPYC_SOLTU	21152.0	—	46855.0	0.17	15938.0	0.73	20924.0	—	20212.0	—	34022.0	—
—	59308.0	0.50	13113.0	—	54391.0	0.03	51040.0	—	49462.0	0.29	41166.0	—
—	33594.0	-0.04	54144.0	-0.41	44621.0	0.59	36848.0	—	36745.0	0.34	61364.0	-0.25
—	—	—	69874.0	—	—	—	53550.0	—	26476.0	0.14	50495.0	0.85
SCAB1_ARATH	52232.0	-0.37	64162.0	-1.26	NF	—	NF	—	NF	—	NF	—
SDHA1_ARATH	5306.0	—	29476.0	—	2363.0	—	11223.0	—	44543.0	—	40732.0	0.43
—	20705.0	—	50151.0	0.07	28329.0	-0.08	31139.0	—	32086.0	-0.44	73750.0	—
—	27994.0	—	54037.0	—	60915.0	—	39401.0	—	50388.0	0.48	76700.0	0.79
—	32538.0	-0.46	49108.0	—	17720.0	-0.79	45717.0	—	—	—	82501.0	0.42
—	—	—	—	—	71899.0	0.18	34920.0	—	—	—	41179.0	—
SUC1_ARATH	NF	—	53.0	—	63389.0	—	NF	—	14608.0	—	NF	—
STP2_ARATH	NF	—	NF	—	23059.0	—	NF	—	16902.0	—	8031.0	—
MYB96_ARATH	NF	—	NF	—	NF	—	NF	—	NF	—	NF	—
PUB13_ARATH	48555.0	—	62220.0	—	37304.1	0.21	44478.1	—	35577.0	0.51	37121.1	0.97
—	50250.0	0.22	33008.0	-0.73	59676.0	-0.04	39001.0	0.90	53555.0	-0.37	68441.0	-0.13
14333_ORYSJ	NF	—	NF	—	NF	—	NF	—	NF	—	NF	—
PSD8A_ARATH	22463.0	—	66259.0	—	12249.0	—	47360.0	—	18856.0	—	64380.0	1.36
—	8305.0	—	—	—	51036.0	-1.24	51649.0	0.62	31880.0	0.32	66290.0	—
—	27506.0	—	—	—	66230.0	—	49806.0	—	8982.0	—	74369.0	—
—	—	—	—	—	26428.0	-0.02	18766.0	—	23097.0	-0.04	73950.0	0.38
—	—	—	—	—	28677.0	*-1.40	44515.0	—	38364.0	0.56	75718.0	-0.08
KCS12_ARATH	59862.0	—	NF	—	35325.0	-0.25	31896.0	—	30958.0	-0.39	NF	—
NCED3_ARATH	37429.0	—	54725.0	—	78234.0	*-2.97	48884.0	*3.56	40341.0	-1.47	79148.0	0.66
ABAH3_ARATH	NF	—	NF	—	NF	—	NF	—	NF	—	NF	—
PYL4_ARATH	NF	—	64731.0	—	36918.0	—	15804.0	—	14619.0	—	40971.0	—

—	—	—	—	—	38747.0	—	21971.0	—	19339.0	0.63	42598.0	—
—	—	—	—	—	43509.0	—	24931.0	-1.22	38723.0	0.39	80026.0	—
—	—	—	—	—	46098.0	—	26662.0	—	—	—	—	—
—	—	—	—	—	51985.0	—	—	—	—	—	—	—
PYL5_ARATH	NF	—	NF	—	32554.0	—	7019.0	—	16691.0	—	62278.0	—
PYL8_ARATH	15351.0	—	65443.0	0.26	52581.0	-1.07	36519.0	—	21321.0	-0.79	56716.0	—
—	51713.0	—	30869.0	—	56051.0	—	40593.0	—	33428.0	—	65307.0	—
—	9070.0	—	31615.0	—	56561.0	—	53179.0	—	46297.0	—	68408.0	*-2.53
—	22672.0	0.33	—	—	69954.0	—	55647.0	—	12507.0	—	74099.0	—
—	60472.0	1.19	—	—	79678.0	—	49611.0	—	43435.0	0.29	77705.0	—
—	—	—	—	—	9053.0	—	28605.0	1.26	26244.0	-0.06	86165.0	—
—	—	—	—	—	51531.0	-0.38	54711.0	—	32619.0	0.71	48696.0	-0.88
—	—	—	—	—	56894.0	0.45	680.0	—	—	—	61932.0	-1.01
—	—	—	—	—	61592.0	—	—	—	—	—	43429.0	—
AI5L5_ARATH	1997.0	—	20640.0	0.72	14599.0	—	15949.0	—	16488.0	—	58616.0	0.18
—	31329.0	0.40	27826.0	—	17403.0	-0.21	21743.0	-0.28	32826.0	—	60111.0	—
—	32392.0	—	59294.0	0.64	36195.0	—	38777.0	—	33412.0	-0.03	79315.0	—
—	60308.0	-1.29	63349.0	—	55221.0	—	39414.0	—	52576.0	-1.79	79821.0	—
—	—	—	—	—	73585.0	—	42416.0	—	35463.0	—	80601.0	0.32
—	—	—	—	—	79680.0	-0.44	47950.0	—	—	—	85753.0	—
—	—	—	—	—	14790.0	0.80	52731.0	—	—	—	71374.0	—
—	—	—	—	—	—	—	55548.0	—	—	—	—	—
—	—	—	—	—	—	—	33036.0	—	—	—	—	—
AGAL_ORYSJ	NF	—	NF	—	NF	—	NF	—	NF	—	NF	—
Q39958_HELAN	NF	—	NF	—	NF	—	NF	—	NF	—	NF	—
PIP21_ARATH	NF	—	NF	—	51261.0	*-3.86	21024.0	—	16268.0	—	66614.0	—
—	—	—	—	—	2192.0	—	—	—	38156.0	—	62374.0	—
—	—	—	—	—	13245.0	—	—	—	—	—	—	—
—	—	—	—	—	22082.0	—	—	—	—	—	—	—
—	—	—	—	—	24320.0	—	—	—	—	—	—	—
—	—	—	—	—	4169.0	—	—	—	—	—	—	—
—	—	—	—	—	17427.0	—	—	—	—	—	—	—
—	—	—	—	—	75869.0	—	—	—	—	—	—	—
—	—	—	—	—	9599.0	—	—	—	—	—	—	—
—	—	—	—	—	57346.0	—	—	—	—	—	—	—
—	—	—	—	—	61247.0	1.33	—	—	—	—	—	—
—	—	—	—	—	71024.0	—	—	—	—	—	—	—
SPE1_ARATH	NF	—	NF	—	NF	—	NF	—	NF	—	NF	—
RQL1_ARATH	27256.0	—	52877.0	—	1732.0	—	41198.0	—	29034.0	—	81069.0	—

—	26364.0	—	28967.0	—	60935.0	—	—	—	—	—	—	—
—	—	—	31648.0	—	79488.0	—	—	—	—	—	—	—
RQL5_ARATH	NF	—	NF	—	15530.0	—	NF	—	37859.0	—	55195.0	—
—	—	—	—	—	50086.0	—	—	—	—	—	83738.0	—
—	—	—	—	—	79234.0	—	—	—	—	—	—	—
SUV3M_ORYSJ	34806.0	—	NF	—	NF	—	NF	—	NF	—	NF	—
ARFG_ARATH	NF	—	NF	—	NF	—	NF	—	NF	—	NF	—
BIG_ARATH	20176.0	—	27359.0	—	21102.0	—	19408.0	—	30289.0	-0.60	34102.0	—
—	21773.0	-0.24	28837.0	0.63	21538.0	—	29644.0	—	40436.0	-0.01	48303.0	*1.71
—	48276.0	0.84	33816.0	0.33	75461.0	-1.19	13916.0	—	7679.0	—	53644.0	*1.91
—	57508.0	—	33972.0	0.11	32151.0	—	—	—	—	—	28180.0	—
—	57508.1	—	49773.0	—	—	—	—	—	—	—	—	—
—	59018.0	—	58797.0	0.53	—	—	—	—	—	—	—	—
—	40523.0	*-1.53	63926.0	—	—	—	—	—	—	—	—	—
—	32488.0	-0.44	65540.0	—	—	—	—	—	—	—	—	—
—	—	—	66590.0	—	—	—	—	—	—	—	—	—
AUX1_ARATH	41000.0	0.13	48388.0	—	22458.0	-0.44	19406.0	-1.19	19013.0	1.09	49241.0	—
—	56453.0	-1.08	59761.0	1.89	23034.0	—	24315.0	—	24409.0	—	67389.0	0.20
—	—	—	67537.0	1.47	42284.0	—	44098.0	-2.11	38423.0	—	73976.0	—
—	—	—	—	—	59703.0	*-1.69	46672.0	—	43511.0	—	81710.0	—
—	—	—	—	—	60652.0	-0.42	57621.0	—	52709.0	—	91781.0	-0.19
—	—	—	—	—	51426.0	—	14507.0	—	—	—	60027.0	0.54
—	—	—	—	—	—	—	12132.0	—	—	—	—	—
Q6IVL3_GOSHI	NF	—	NF	—	NF	—	NF	—	NF	—	NF	—
CDPKN_ARATH	NF	—	NF	—	NF	—	NF	—	NF	—	NF	—
CB22_ARATH	NF	—	NF	—	NF	—	NF	—	NF	—	NF	—
CB3_ARATH	33647.0	0.88	29531.0	—	28158.0	—	11689.0	—	21075.0	—	50686.0	—
CA4_ARATH	40546.0	*-1.89	12301.0	—	43886.0	—	NF	—	NF	—	8266.0	—
—	40113.0	*-1.70	20719.0	—	9244.0	—	—	—	—	—	42550.0	—
—	28496.0	—	—	—	63696.0	1.01	—	—	—	—	45962.0	—
—	—	—	—	—	13901.0	—	—	—	—	—	47470.0	—
—	—	—	—	—	—	—	—	—	—	—	60006.0	0.53
—	—	—	—	—	—	—	—	—	—	—	88113.0	0.04
CSP2_ARATH	NF	—	NF	—	NF	—	NF	—	NF	—	NF	—
DRE1A_ARATH	20891.0	—	69584.0	—	NF	—	NF	—	NF	—	NF	—
DRE1B_ARATH	31625.0	—	31046.0	—	NF	—	NF	—	NF	—	NF	—
—	—	—	39486.0	—	—	—	—	—	—	—	—	—
—	3975.0	—	46882.0	—	—	—	—	—	—	—	—	—
—	—	—	46882.0	—	—	—	—	—	—	—	—	—

DRE1C_ARATH	NF	—	NF	—	49962.0	—	2184.0	—	26626.0	—	35024.0	—
—	—	—	—	—	—	—	4186.0	—	40833.0	-1.07	48587.0	1.09
DRE2A_ARATH	NF	—	32555.0	—	57685.0	—	37216.0	—	53037.0	—	65485.0	—
—	—	—	48161.0	—	68844.0	—	1348.0	—	40862.0	*2.21	51543.0	—
—	—	—	—	—	36498.0	—	2488.0	—	53836.0	*1.99	90226.0	-0.41
COR47_ARATH	42834.0	*2.10	27118.0	0.87	3433.0	—	NF	—	45038.0	—	49053.0	—
—	—	—	60204.0	—	NF	—	—	—	—	—	—	—
RZF1_ARATH	27763.1	—	35403.0	—	19813.0	—	NF	—	NF	—	NF	—
SDIR1_ARATH	56824.0	*-1.96	35045.0	-0.27	20889.0	—	55882.0	—	30086.0	—	43117.0	—
—	20382.0	—	44788.0	0.39	60311.0	—	1710.0	—	33144.0	—	65865.0	—
—	43020.0	-0.84	48693.0	—	66070.0	—	35587.0	—	10214.0	—	31466.0	—
—	—	—	—	—	18538.0	—	29054.0	—	—	—	29667.0	—
A0A1S2Z179_CICAR	NF	—	NF	—	NF	—	NF	—	NF	—	NF	—
SINA3_ARATH	51265.0	-0.26	26018.0	—	33582.0	0.41	42684.0	-0.52	43359.0	0.19	87037.0	-0.73
—	32570.0	-0.01	34484.0	—	18831.0	-0.58	46118.0	0.69	43690.0	-0.17	77433.0	-0.84
—	47711.0	-1.47	53934.0	0.34	—	—	—	—	—	—	—	—
—	—	—	60529.0	0.55	—	—	—	—	—	—	—	—
ELP1_ARATH	39468.0	-0.21	29575.0	0.43	51648.0	—	52021.0	—	Cluster-20592.0	—	44084.0	—
—	9730.0	—	—	—	42238.1	0.17	52021.2	-0.40	Cluster-43395.0	-0.13	82243.0	-1.38
—	35892.0	-0.10	—	—	42238.0	—	52021.1	—	—	—	38666.0	—
—	62044.0	-0.36	—	—	—	—	—	—	—	—	—	—
ABI4_ARATH	NF	—	NF	—	NF	—	NF	—	NF	—	NF	—
WIN1_ARATH	NF	—	45954.0	—	28899.0	—	1632.0	—	50935.0	—	NF	—
—	—	—	—	—	3476.0	—	19788.0	—	25222.0	—	—	—
ALFC7_ARATH	NF	—	NF	—	NF	—	NF	—	NF	—	NF	—
GOLS3_ARATH	NF	—	NF	—	NF	—	NF	—	NF	—	NF	—
GSTFA_ARATH	NF	—	41497.0	—	NF	—	NF	—	NF	—	NF	—
GSTUJ_ARATH	NF	—	NF	—	NF	—	NF	—	NF	—	NF	—
HPR1_ARATH	17372.0	—	36501.0	—	73490.0	—	364.0	—	9949.0	—	86714.0	—
—	60671.0	—	57114.0	—	56851.0	—	—	—	35920.0	—	—	—
GLPK_ARATH	NF	—	NF	—	NF	—	NF	—	NF	—	48301.0	—
GLYR1_ARATH	35836.0	—	37301.0	—	63668.0	—	57557.0	—	39568.0	-0.67	81750.0	—
—	—	—	—	—	1141.0	—	31059.0	—	8548.0	—	56776.0	—
—	—	—	—	—	13446.0	—	—	—	13094.0	—	5765.0	—
—	—	—	—	—	46780.0	0.01	—	—	—	—	64278.0	—
—	—	—	—	—	52601.0	—	—	—	—	—	—	—
SLAC1_ARATH	7432.0	—	NF	—	63580.0	*-2.65	25632.0	—	35818.0	0.87	NF	—

AHK1_ARATH	28013.0	—	41916.0	—	24650.0	—	18000.0	—	46685.0	—	77788.0	—
—	63284.0	-0.20	4422.0	—	49294.1	—	40232.0	—	19410.0	0.04	81623.0	—
—	—	—	—	—	24650.1	-0.98	—	—	—	—	6718.0	—
AHK3_ARATH	NF	—	NF	—	NF	—	NF	—	NF	—	NF	—
AHK4_ARATH	37288.0	1.64	19175.0	—	59014.0	1.17	32554.0	-1.41	23810.0	-0.56	74833.0	-0.59
—	40197.0	—	41092.0	1.63	—	—	—	—	—	—	—	—
—	56831.0	1.46	44187.0	1.74	—	—	—	—	—	—	—	—
—	39205.0	—	—	—	—	—	—	—	—	—	—	—
AHP5_ARATH	33135.0	0.44	44232.0	0.31	3642.0	—	NF	—	16727.0	—	42384.0	—
—	30617.0	0.06	67132.0	-0.79	—	—	—	—	—	—	—	—
YUC6_ARATH	NF	—	NF	—	45456.0	—	41005.0	—	50129.0	—	22995.0	—
—	—	—	—	—	8311.0	—	—	—	9948.0	—	—	—
—	—	—	—	—	54283.0	—	—	—	—	—	—	—
B6UH99_MAIZE	NF	—	NF	—	NF	—	NF	—	NF	—	NF	—
Q9M0X3_ARATH	NF	—	NF	—	NF	—	NF	—	NF	—	NF	—
F4JQF1_ARATH	NF	—	NF	—	NF	—	NF	—	NF	—	NF	—
LEA46_ARATH	6646.0	—	11690.0	—	11294.0	—	NF	—	NF	—	78366.0	—
—	6725.0	—	52317.0	1.09	—	—	—	—	—	—	—	—
—	32615.0	—	10766.0	—	—	—	—	—	—	—	—	—
LEA41_ARATH	NF	—	NF	—	NF	—	NF	—	NF	—	NF	—
LACS2_ARATH	37578.0	0.74	44475.0	—	74518.0	—	9880.0	—	23105.0	—	NF	—
—	—	—	—	—	63173.0	—	—	—	—	—	—	—
MED25_ARATH	40951.0	-1.21	62487.0	0.49	40206.0	—	37187.0	—	38565.0	—	29020.0	—
VDAC3_ARATH	NF	—	NF	—	NF	—	NF	—	NF	—	NF	—
PUMP1_ARATH	62786.0	—	48804.0	—	33421.0	—	22483.0	—	13619.0	—	57109.0	—
—	8716.0	—	55602.0	—	54638.0	—	36943.0	—	30795.0	0.35	46907.0	—
—	43319.0	0.28	65832.0	-0.04	27543.0	-0.13	41493.0	1.18	—	—	48231.1	0.27
M2K1_ARATH	NF	—	NF	—	NF	—	NF	—	NF	—	NF	—
MP3K2_ARATH	NF	—	NF	—	NF	—	NF	—	NF	—	NF	—
NAC52_ARATH	NF	—	NF	—	52816.0	-0.10	52054.0	—	35797.0	0.78	33176.0	*2.00
—	—	—	—	—	—	—	559.0	—	46290.0	0.97	80639.0	—
NAC40_ARATH	37788.0	—	NF	—	23175.0	-0.58	14773.0	—	43811.0	-1.12	86643.0	1.00
—	—	—	—	—	—	—	43581.0	0.82	—	—	—	—
NAC48_ORYSJ	56568.0	0.42	32595.0	—	NF	—	NF	—	NF	—	NF	—
NAC53_ARATH	27987.0	-1.47	53278.0	0.20	43602.0	—	25392.0	—	24549.0	-0.88	67740.0	—
—	—	—	—	—	75638.0	-1.12	46806.0	0.06	47866.0	-0.03	70886.0	-0.14
NAC62_ARATH	NF	—	NF	—	NF	—	NF	—	NF	—	NF	—
NAC56_ARATH	28107.0	—	48011.0	*2.90	18762.0	—	29455.0	—	15242.0	—	56652.0	—
—	51001.0	0.00	—	—	48643.0	—	49666.0	—	50239.0	—	—	—

—	—	—	—	—	57201.0	—	—	—	—	—	—	—
NDUS1_ARATH	NF	—	NF	—	NF	—	NF	—	NF	—	NF	—
AKRC9_ARATH	10817.0	—	29343.0	-0.22	2082.0	—	23710.0	—	44077.0	—	37016.0	—
—	44870.0	0.68	39628.0	—	46868.0	—	52521.0	—	28509.0	0.71	85265.0	—
—	—	—	—	—	63167.0	-0.11	37228.0	—	—	—	87849.0	—
—	—	—	—	—	—	—	—	—	—	—	69958.0	1.53
NPC4_ARATH	NF	—	NF	—	73777.0	—	35738.0	1.57	14807.0	—	66947.0	0.00
—	—	—	—	—	63382.0	—	49197.0	—	16543.0	—	87126.0	—
Q5U9M2_ORYSJ	NF	—	NF	—	NF	—	NF	—	NF	—	NF	—
Q0J265_ORYSJ	NF	—	NF	—	NF	—	NF	—	NF	—	NF	—
PER33_ARATH	NF	—	NF	—	NF	—	NF	—	NF	—	NF	—
PER34_ARATH	NF	—	NF	—	NF	—	NF	—	NF	—	NF	—
PGL1A_ARATH	41556.0	—	34846.0	*-3.47	14163.0	0.72	54107.0	—	37867.0	0.64	88780.0	—
—	45791.0	0.46	47604.0	—	—	—	—	—	—	—	—	—
RFS5_ARATH	33272.0	1.14	29161.0	-1.07	76965.0	*-1.81	42554.0	—	31224.0	0.25	24183.0	—
ABI5_ARATH	NF	—	NF	—	NF	—	NF	—	NF	—	NF	—
AB1K1_ARATH	NF	—	NF	—	18052.0	0.63	18851.0	—	21916.0	0.59	85765.0	—
DTX43_ARATH	38777.0	-0.51	31816.0	1.31	74815.0	—	53199.0	—	NF	—	35781.0	0.36
ERD15_ARATH	51610.0	—	56471.0	0.83	49900.0	—	11294.0	—	12794.0	—	NF	—
—	45143.0	*2.03	23659.0	—	53720.0	—	21884.0	—	—	—	—	—
—	—	—	—	—	54119.0	—	33027.0	—	—	—	—	—
MET1_ARATH	11490.0	—	35467.0	—	53131.0	—	3381.0	—	42413.0	—	52721.0	—
—	4644.0	—	47231.0	—	67555.0	—	1241.0	—	8864.0	—	64336.0	—
—	59675.0	1.68	62256.0	—	77088.0	—	—	—	—	—	—	—
—	—	—	62817.0	—	—	—	—	—	—	—	—	—
Q7XJ04_ORYSJ	NF	—	NF	—	NF	—	NF	—	NF	—	NF	—
PPOX1_ARATH	NF	—	NF	—	3876.0	—	NF	—	NF	—	61957.0	—
KPYC_SOLTU	21152.0	—	46855.0	0.17	15938.0	0.73	20924.0	—	20212.0	—	34022.0	—
—	59308.0	0.50	13113.0	—	54391.0	0.03	51040.0	—	49462.0	0.29	41166.0	—
—	33594.0	-0.04	54144.0	-0.41	44621.0	0.59	36848.0	—	36745.0	0.34	61364.0	-0.25
—	—	—	69874.0	—	—	—	53550.0	—	26476.0	0.14	50495.0	0.85
SCAB1_ARATH	52232.0	-0.37	64162.0	-1.26	NF	—	NF	—	NF	—	NF	—
SDHA1_ARATH	5306.0	—	29476.0	—	2363.0	—	11223.0	—	44543.0	—	40732.0	0.43
—	20705.0	—	50151.0	0.07	28329.0	-0.08	31139.0	—	32086.0	-0.44	73750.0	—
—	27994.0	—	54037.0	—	60915.0	—	39401.0	—	50388.0	0.48	76700.0	0.79
—	32538.0	-0.46	49108.0	—	17720.0	-0.79	45717.0	—	—	—	82501.0	0.42
—	—	—	—	—	71899.0	0.18	34920.0	—	—	—	41179.0	—
SUC1_ARATH	NF	—	53.0	—	63389.0	—	NF	—	14608.0	—	NF	—
STP2_ARATH	NF	—	NF	—	23059.0	—	NF	—	16902.0	—	8031.0	—

MYB96_ARATH	NF	—	NF	—	NF	—	NF	—	NF	—	NF	—
PUB13_ARATH	48555.0	—	62220.0	—	37304.1	0.21	44478.1	—	35577.0	0.51	37121.1	0.97
—	50250.0	0.22	33008.0	-0.73	59676.0	-0.04	39001.0	0.90	53555.0	-0.37	68441.0	-0.13
14333_ORYSJ	NF	—	NF	—	NF	—	NF	—	NF	—	NF	—
PSD8A_ARATH	22463.0	—	66259.0	—	12249.0	—	47360.0	—	18856.0	—	64380.0	1.36
—	8305.0	—	—	—	51036.0	-1.24	51649.0	0.62	31880.0	0.32	66290.0	—
—	27506.0	—	—	—	66230.0	—	49806.0	—	8982.0	—	74369.0	—
—	—	—	—	—	26428.0	-0.02	18766.0	—	23097.0	-0.04	73950.0	0.38
—	—	—	—	—	28677.0	*-1.40	44515.0	—	38364.0	0.56	75718.0	-0.08
KCS12_ARATH	59862.0	—	NF	—	35325.0	-0.25	31896.0	—	30958.0	-0.39	NF	—
NCED3_ARATH	37429.0	—	54725.0	—	78234.0	*-2.97	48884.0	*3.56	40341.0	-1.47	79148.0	0.66
ABAH3_ARATH	NF	—	NF	—	NF	—	NF	—	NF	—	NF	—
PYL4_ARATH	NF	—	64731.0	—	36918.0	—	15804.0	—	14619.0	—	40971.0	—
—	—	—	—	—	38747.0	—	21971.0	—	19339.0	0.63	42598.0	—
—	—	—	—	—	43509.0	—	24931.0	-1.22	38723.0	0.39	80026.0	—
—	—	—	—	—	46098.0	—	26662.0	—	—	—	—	—
—	—	—	—	—	51985.0	—	—	—	—	—	—	—
PYL5_ARATH	NF	—	NF	—	32554.0	—	7019.0	—	16691.0	—	62278.0	—
PYL8_ARATH	15351.0	—	65443.0	0.26	52581.0	-1.07	36519.0	—	21321.0	-0.79	56716.0	—
—	51713.0	—	30869.0	—	56051.0	—	40593.0	—	33428.0	—	65307.0	—
—	9070.0	—	31615.0	—	56561.0	—	53179.0	—	46297.0	—	68408.0	*-2.53
—	22672.0	0.33	—	—	69954.0	—	55647.0	—	12507.0	—	74099.0	—
—	60472.0	1.19	—	—	79678.0	—	49611.0	—	43435.0	0.29	77705.0	—
—	—	—	—	—	9053.0	—	28605.0	1.26	26244.0	-0.06	86165.0	—
—	—	—	—	—	51531.0	-0.38	54711.0	—	32619.0	0.71	48696.0	-0.88
—	—	—	—	—	56894.0	0.45	680.0	—	—	—	61932.0	-1.01
—	—	—	—	—	61592.0	—	—	—	—	—	43429.0	—
AI5L5_ARATH	1997.0	—	20640.0	0.72	14599.0	—	15949.0	—	16488.0	—	58616.0	0.18
—	31329.0	0.40	27826.0	—	17403.0	-0.21	21743.0	-0.28	32826.0	—	60111.0	—
—	32392.0	—	59294.0	0.64	36195.0	—	38777.0	—	33412.0	-0.03	79315.0	—
—	60308.0	-1.29	63349.0	—	55221.0	—	39414.0	—	52576.0	-1.79	79821.0	—
—	—	—	—	—	73585.0	—	42416.0	—	35463.0	—	80601.0	0.32
—	—	—	—	—	79680.0	-0.44	47950.0	—	—	—	85753.0	—
—	—	—	—	—	14790.0	0.80	52731.0	—	—	—	71374.0	—
—	—	—	—	—	—	—	55548.0	—	—	—	—	—
—	—	—	—	—	—	—	33036.0	—	—	—	—	—
AGAL_ORYSJ	NF	—	NF	—	NF	—	NF	—	NF	—	NF	—

Q39958_HELAN	NF	—	NF	—	NF	—	NF	—	NF	—	NF	—
PIP21_ARATH	NF	—	NF	—	51261.0	*-3.86	21024.0	—	16268.0	—	66614.0	—
—	—	—	—	—	2192.0	—	—	—	38156.0	—	62374.0	—
—	—	—	—	—	13245.0	—	—	—	—	—	—	—
—	—	—	—	—	22082.0	—	—	—	—	—	—	—
—	—	—	—	—	24320.0	—	—	—	—	—	—	—
—	—	—	—	—	4169.0	—	—	—	—	—	—	—
—	—	—	—	—	17427.0	—	—	—	—	—	—	—
—	—	—	—	—	75869.0	—	—	—	—	—	—	—
—	—	—	—	—	9599.0	—	—	—	—	—	—	—
—	—	—	—	—	57346.0	—	—	—	—	—	—	—
—	—	—	—	—	61247.0	1.33	—	—	—	—	—	—
—	—	—	—	—	71024.0	—	—	—	—	—	—	—
SPE1_ARATH	NF	—	NF	—	NF	—	NF	—	NF	—	NF	—
RQL1_ARATH	27256.0	—	52877.0	—	1732.0	—	41198.0	—	29034.0	—	81069.0	—
—	26364.0	—	28967.0	—	60935.0	—	—	—	—	—	—	—
—	—	—	31648.0	—	79488.0	—	—	—	—	—	—	—
RQL5_ARATH	NF	—	NF	—	15530.0	—	NF	—	37859.0	—	55195.0	—
—	—	—	—	—	50086.0	—	—	—	—	—	83738.0	—
—	—	—	—	—	79234.0	—	—	—	—	—	—	—
SUV3M_ORYSJ	34806.0	—	NF	—	NF	—	NF	—	NF	—	NF	—
ARFG_ARATH	NF	—	NF	—	NF	—	NF	—	NF	—	NF	—
BIG_ARATH	20176.0	—	27359.0	—	21102.0	—	19408.0	—	30289.0	-0.60	34102.0	—
—	21773.0	-0.24	28837.0	0.63	21538.0	—	29644.0	—	40436.0	-0.01	48303.0	*1.71
—	48276.0	0.84	33816.0	0.33	75461.0	-1.19	13916.0	—	7679.0	—	53644.0	*1.91
—	57508.0	—	33972.0	0.11	32151.0	—	—	—	—	—	28180.0	—
—	57508.1	—	49773.0	—	—	—	—	—	—	—	—	—
—	59018.0	—	58797.0	0.53	—	—	—	—	—	—	—	—
—	40523.0	*-1.53	63926.0	—	—	—	—	—	—	—	—	—
—	32488.0	-0.44	65540.0	—	—	—	—	—	—	—	—	—
—	—	—	66590.0	—	—	—	—	—	—	—	—	—
AUX1_ARATH	41000.0	0.13	48388.0	—	22458.0	-0.44	19406.0	-1.19	19013.0	1.09	49241.0	—
—	56453.0	-1.08	59761.0	1.89	23034.0	—	24315.0	—	24409.0	—	67389.0	0.20
—	—	—	67537.0	1.47	42284.0	—	44098.0	-2.11	38423.0	—	73976.0	—
—	—	—	—	—	59703.0	*-1.69	46672.0	—	43511.0	—	81710.0	—
—	—	—	—	—	60652.0	-0.42	57621.0	—	52709.0	—	91781.0	-0.19
—	—	—	—	—	51426.0	—	14507.0	—	—	—	60027.0	0.54

—	—	—	—	—	—	—	12132.0	—	—	—	—	—
Q6IVL3_GOSHI	NF	—	NF	—	NF	—	NF	—	NF	—	NF	—
CDPKN_ARATH	NF	—	NF	—	NF	—	NF	—	NF	—	NF	—
CB22_ARATH	NF	—	NF	—	NF	—	NF	—	NF	—	NF	—
CB3_ARATH	33647.0	0.88	29531.0	—	28158.0	—	11689.0	—	21075.0	—	50686.0	—
CA4_ARATH	40546.0	*-1.89	12301.0	—	43886.0	—	NF	—	NF	—	8266.0	—
—	40113.0	*-1.70	20719.0	—	9244.0	—	—	—	—	—	42550.0	—
—	28496.0	—	—	—	63696.0	1.01	—	—	—	—	45962.0	—
—	—	—	—	—	13901.0	—	—	—	—	—	47470.0	—
—	—	—	—	—	—	—	—	—	—	—	60006.0	0.53
—	—	—	—	—	—	—	—	—	—	—	88113.0	0.04
CSP2_ARATH	NF	—	NF	—	NF	—	NF	—	NF	—	NF	—
DRE1A_ARATH	20891.0	—	69584.0	—	NF	—	NF	—	NF	—	NF	—
DRE1B_ARATH	31625.0	—	31046.0	—	NF	—	NF	—	NF	—	NF	—
—	—	—	39486.0	—	—	—	—	—	—	—	—	—
—	3975.0	—	46882.0	—	—	—	—	—	—	—	—	—
—	—	—	46882.0	—	—	—	—	—	—	—	—	—
DRE1C_ARATH	NF	—	NF	—	49962.0	—	2184.0	—	26626.0	—	35024.0	—
—	—	—	—	—	—	—	4186.0	—	40833.0	-1.07	48587.0	1.09
DRE2A_ARATH	NF	—	32555.0	—	57685.0	—	37216.0	—	53037.0	—	65485.0	—
—	—	—	48161.0	—	68844.0	—	1348.0	—	40862.0	*2.21	51543.0	—
—	—	—	—	—	36498.0	—	2488.0	—	53836.0	*1.99	90226.0	-0.41
COR47_ARATH	42834.0	*2.10	27118.0	0.87	3433.0	—	NF	—	45038.0	—	49053.0	—
—	—	—	60204.0	—	NF	—	—	—	—	—	—	—
RZF1_ARATH	27763.1	—	35403.0	—	19813.0	—	NF	—	NF	—	NF	—
SDIR1_ARATH	56824.0	*-1.96	35045.0	-0.27	20889.0	—	55882.0	—	30086.0	—	43117.0	—
—	20382.0	—	44788.0	0.39	60311.0	—	1710.0	—	33144.0	—	65865.0	—
—	43020.0	-0.84	48693.0	—	66070.0	—	35587.0	—	10214.0	—	31466.0	—
—	—	—	—	—	18538.0	—	29054.0	—	—	—	29667.0	—
A0A1S2Z179_CICAR	NF	—	NF	—	NF	—	NF	—	NF	—	NF	—
SINA3_ARATH	51265.0	-0.26	26018.0	—	33582.0	0.41	42684.0	-0.52	43359.0	0.19	87037.0	-0.73
—	32570.0	-0.01	34484.0	—	18831.0	-0.58	46118.0	0.69	43690.0	-0.17	77433.0	-0.84
—	47711.0	-1.47	53934.0	0.34	—	—	—	—	—	—	—	—
—	—	—	60529.0	0.55	—	—	—	—	—	—	—	—
ELP1_ARATH	39468.0	-0.21	29575.0	0.43	51648.0	—	52021.0	—	20592.0	—	44084.0	—
—	9730.0	—	—	—	42238.1	0.17	52021.2	-0.40	43395.0	-0.13	82243.0	-1.38
—	35892.0	-0.10	—	—	42238.0	—	52021.1	—	—	—	38666.0	—
—	62044.0	-0.36	—	—	—	—	—	—	—	—	—	—
ABI4_ARATH	NF	—	NF	—	NF	—	NF	—	NF	—	NF	—

WIN1_ARATH	NF	—	45954.0	—	28899.0	—	1632.0	—	50935.0	—	NF	—
—	—	—	—	—	3476.0	—	19788.0	—	25222.0	—	—	—
ALFC7_ARATH	NF	—	NF	—	NF	—	NF	—	NF	—	NF	—
GOLS3_ARATH	NF	—	NF	—	NF	—	NF	—	NF	—	NF	—
GSTFA_ARATH	NF	—	41497.0	—	NF	—	NF	—	NF	—	NF	—
GSTUJ_ARATH	NF	—	NF	—	NF	—	NF	—	NF	—	NF	—
HPR1_ARATH	17372.0	—	36501.0	—	73490.0	—	364.0	—	9949.0	—	86714.0	—
—	60671.0	—	57114.0	—	56851.0	—	—	—	35920.0	—	—	—
GLPK_ARATH	NF	—	NF	—	NF	—	NF	—	NF	—	48301.0	—
GLYR1_ARATH	35836.0	—	37301.0	—	63668.0	—	57557.0	—	39568.0	-0.67	81750.0	—
—	—	—	—	—	1141.0	—	31059.0	—	8548.0	—	56776.0	—
—	—	—	—	—	13446.0	—	—	—	13094.0	—	5765.0	—
—	—	—	—	—	46780.0	0.01	—	—	—	—	64278.0	—
—	—	—	—	—	52601.0	—	—	—	—	—	—	—
						*-						
SLAC1_ARATH	7432.0	—	NF	—	63580.0	2.65	25632.0	—	35818.0	0.87	NF	—
AHK1_ARATH	28013.0	—	41916.0	—	24650.0	—	18000.0	—	46685.0	—	77788.0	—
—	63284.0	-0.20	4422.0	—	49294.1	—	40232.0	—	19410.0	0.04	81623.0	—
—	—	—	—	—	24650.1	-0.98	—	—	—	—	6718.0	—
AHK3_ARATH	NF	—	NF	—	NF	—	NF	—	NF	—	NF	—
AHK4_ARATH	37288.0	1.64	19175.0	—	59014.0	1.17	32554.0	-1.41	23810.0	-0.56	74833.0	-0.59
—	40197.0	—	41092.0	1.63	—	—	—	—	—	—	—	—
—	56831.0	1.46	44187.0	1.74	—	—	—	—	—	—	—	—
—	39205.0	—	—	—	—	—	—	—	—	—	—	—
AHP5_ARATH	33135.0	0.44	44232.0	0.31	3642.0	—	NF	—	16727.0	—	42384.0	—
—	30617.0	0.06	67132.0	-0.79	—	—	—	—	—	—	—	—
YUC6_ARATH	NF	—	NF	—	45456.0	—	41005.0	—	50129.0	—	22995.0	—
—	—	—	—	—	8311.0	—	—	—	9948.0	—	—	—
—	—	—	—	—	54283.0	—	—	—	—	—	—	—
B6UH99_MAIZE	NF	—	NF	—	NF	—	NF	—	NF	—	NF	—
Q9M0X3_ARATH	NF	—	NF	—	NF	—	NF	—	NF	—	NF	—
F4JQF1_ARATH	NF	—	NF	—	NF	—	NF	—	NF	—	NF	—
LEA46_ARATH	6646.0	—	11690.0	—	11294.0	—	NF	—	NF	—	78366.0	—
—	6725.0	—	52317.0	1.09	—	—	—	—	—	—	—	—
—	32615.0	—	10766.0	—	—	—	—	—	—	—	—	—
LEA41_ARATH	NF	—	NF	—	NF	—	NF	—	NF	—	NF	—
LACS2_ARATH	37578.0	0.74	44475.0	—	74518.0	—	9880.0	—	23105.0	—	NF	—
—	—	—	—	—	63173.0	—	—	—	—	—	—	—
MED25_ARATH	40951.0	-1.21	62487.0	0.49	40206.0	—	37187.0	—	38565.0	—	29020.0	—

VDAC3_ARATH	NF	—	NF	—	NF	—	NF	—	NF	—	NF	—
PUMP1_ARATH	62786.0	—	48804.0	—	33421.0	—	22483.0	—	13619.0	—	57109.0	—
—	8716.0	—	55602.0	—	54638.0	—	36943.0	—	30795.0	0.35	46907.0	—
—	43319.0	0.28	65832.0	-0.04	27543.0	-0.13	41493.0	1.18	—	—	48231.1	0.27
M2K1_ARATH	NF	—	NF	—	NF	—	NF	—	NF	—	NF	—
MP3K2_ARATH	NF	—	NF	—	NF	—	NF	—	NF	—	NF	—
NAC52_ARATH	NF	—	NF	—	52816.0	-0.10	52054.0	—	35797.0	0.78	33176.0	*2.00
—	—	—	—	—	—	—	559.0	—	46290.0	0.97	80639.0	—
NAC40_ARATH	37788.0	—	NF	—	23175.0	-0.58	14773.0	—	43811.0	-1.12	86643.0	1.00
—	—	—	—	—	—	—	43581.0	0.82	—	—	—	—
NAC48_ORYSJ	56568.0	0.42	32595.0	—	NF	—	NF	—	NF	—	NF	—
NAC53_ARATH	27987.0	-1.47	53278.0	0.20	43602.0	—	25392.0	—	24549.0	-0.88	67740.0	—
—	—	—	—	—	75638.0	-1.12	46806.0	0.06	47866.0	-0.03	70886.0	-0.14
NAC62_ARATH	NF	—	NF	—	NF	—	NF	—	NF	—	NF	—
NAC56_ARATH	28107.0	—	48011.0	*2.90	18762.0	—	29455.0	—	15242.0	—	56652.0	—
—	51001.0	0.00	—	—	48643.0	—	49666.0	—	50239.0	—	—	—
—	—	—	—	—	57201.0	—	—	—	—	—	—	—
NDUS1_ARATH	NF	—	NF	—	NF	—	NF	—	NF	—	NF	—
AKRC9_ARATH	10817.0	—	29343.0	-0.22	2082.0	—	23710.0	—	44077.0	—	37016.0	—
—	44870.0	0.68	39628.0	—	46868.0	—	52521.0	—	28509.0	0.71	85265.0	—
—	—	—	—	—	63167.0	-0.11	37228.0	—	—	—	87849.0	—
—	—	—	—	—	—	—	—	—	—	—	69958.0	1.53
NPC4_ARATH	NF	—	NF	—	73777.0	—	35738.0	1.57	14807.0	—	66947.0	0.00
—	—	—	—	—	63382.0	—	49197.0	—	16543.0	—	87126.0	—
Q5U9M2_ORYSJ	NF	—	NF	—	NF	—	NF	—	NF	—	NF	—
Q0J265_ORYSJ	NF	—	NF	—	NF	—	NF	—	NF	—	NF	—
PER33_ARATH	NF	—	NF	—	NF	—	NF	—	NF	—	NF	—
PER34_ARATH	NF	—	NF	—	NF	—	NF	—	NF	—	NF	—
PGL1A_ARATH	41556.0	—	34846.0	*-3.47	14163.0	0.72	54107.0	—	37867.0	0.64	88780.0	—
—	45791.0	0.46	47604.0	—	—	—	—	—	—	—	—	—
RFS5_ARATH	33272.0	1.14	29161.0	-1.07	76965.0	*-1.81	42554.0	—	31224.0	0.25	24183.0	—
ABI5_ARATH	NF	—	NF	—	NF	—	NF	—	NF	—	NF	—
AB1K1_ARATH	NF	—	NF	—	18052.0	0.63	18851.0	—	21916.0	0.59	85765.0	—
DTX43_ARATH	38777.0	-0.51	31816.0	1.31	74815.0	—	53199.0	—	NF	—	35781.0	0.36
ERD15_ARATH	51610.0	—	56471.0	0.83	49900.0	—	11294.0	—	12794.0	—	NF	—
—	45143.0	*2.03	23659.0	—	53720.0	—	21884.0	—	—	—	—	—
—	—	—	—	—	54119.0	—	33027.0	—	—	—	—	—
MET1_ARATH	11490.0	—	35467.0	—	53131.0	—	3381.0	—	42413.0	—	52721.0	—

—	4644.0	—	47231.0	—	67555.0	—	1241.0	—	8864.0	—	64336.0	—
—	59675.0	1.68	62256.0	—	77088.0	—	—	—	—	—	—	—
—	—	—	62817.0	—	—	—	—	—	—	—	—	—
Q7XJ04_ORYSJ	NF	—	NF	—	NF	—	NF	—	NF	—	NF	—
PPOX1_ARATH	NF	—	NF	—	3876.0	—	NF	—	NF	—	61957.0	—
KPYC_SOLTU	21152.0	—	46855.0	0.17	15938.0	0.73	20924.0	—	20212.0	—	34022.0	—
—	59308.0	0.50	13113.0	—	54391.0	0.03	51040.0	—	49462.0	0.29	41166.0	—
—	33594.0	-0.04	54144.0	-0.41	44621.0	0.59	36848.0	—	36745.0	0.34	61364.0	-0.25
—	—	—	69874.0	—	—	—	53550.0	—	26476.0	0.14	50495.0	0.85
SCAB1_ARATH	52232.0	-0.37	64162.0	-1.26	NF	—	NF	—	NF	—	NF	—
SDHA1_ARATH	5306.0	—	29476.0	—	2363.0	—	11223.0	—	44543.0	—	40732.0	0.43
—	20705.0	—	50151.0	0.07	28329.0	-0.08	31139.0	—	32086.0	-0.44	73750.0	—
—	27994.0	—	54037.0	—	60915.0	—	39401.0	—	50388.0	0.48	76700.0	0.79
—	32538.0	-0.46	49108.0	—	17720.0	-0.79	45717.0	—	—	—	82501.0	0.42
—	—	—	—	—	71899.0	0.18	34920.0	—	—	—	41179.0	—
SUC1_ARATH	NF	—	53.0	—	63389.0	—	NF	—	14608.0	—	NF	—
STP2_ARATH	NF	—	NF	—	23059.0	—	NF	—	16902.0	—	8031.0	—
MYB96_ARATH	NF	—	NF	—	NF	—	NF	—	NF	—	NF	—
PUB13_ARATH	48555.0	—	62220.0	—	37304.1	0.21	44478.1	—	35577.0	0.51	37121.1	0.97
—	50250.0	0.22	33008.0	-0.73	59676.0	-0.04	39001.0	0.90	53555.0	-0.37	68441.0	-0.13
